# Human *CIDEC* transgene improves lipid metabolism and protects against high-fat diet–induced glucose intolerance in mice

**DOI:** 10.1016/j.jbc.2022.102347

**Published:** 2022-08-11

**Authors:** Abhishek Gupta, Bijinu Balakrishnan, Shakun Karki, Mark Slayton, Sukanta Jash, Sayani Banerjee, Tan Hooi Min Grahn, Srikarthika Jambunathan, Sarah Disney, Hebaallaha Hussein, Dong Kong, Bradford B. Lowell, Purushothaman Natarajan, Umesh K. Reddy, Noyan Gokce, Vishva M. Sharma, Vishwajeet Puri

**Affiliations:** 1 Department of Biomedical Sciences and Diabetes Institute, Heritage College of Osteopathic Medicine, Ohio University, Athens, Ohio, USA; 2 Evans Department of Medicine and Whitaker Cardiovascular Institute, Boston University School of Medicine, Boston, Massachusetts, USA; 3 Alpert Medical School of Brown University, Brown University, Providence, Rhode Island, USA; 4 Division of Molecular Medicine and Gene Therapy, Lund Stem Cell Center, Lund University Hospital, Lund, Sweden; 5 Department of Medicine, Boston University School of Medicine, Boston, Massachusetts, USA; 6 Division of Endocrinology, Department of Pediatrics, F.M. Kirby Neurobiology Center, Boston Children’s Hospital and Harvard Medical School, Boston, Massachusetts, USA; 7 Division of Endocrinology, Diabetes, and Metabolism, Department of Medicine, Beth Israel Deaconess Medical Center, Boston, Massachusetts, USA; 8 Program in Neuroscience, Harvard Medical School, Boston, Massachusetts, USA; 9 Department of Biology, West Virginia State University, Institute, West Virginia, USA

**Keywords:** CGI-58, metabolism, Cidec, lipids, lipid droplets, diabetes, obesity, FSP27, BSA, bovine serum albumin, cDNA, complementary DNA, DEG, differentially expressed gene, FA, fatty acid, FFA, free fatty acid, GTT, glucose tolerance test, HDL, high-density lipoproteins, HFD, high fat diet, HRP, horseradish peroxidase, ITT, insulin tolerance test, LDL, low-density lipoprotein, PBST, PBS with Tween-20, SAT, subcutaneous adipose tissue, TBS, Tris-buffered saline, TG, triglyceride, VAT, visceral adipose tissue

## Abstract

Cell death–inducing DNA fragmentation factor-like effector C (CIDEC) expression in adipose tissue positively correlates with insulin sensitivity in obese humans. Further, E186X, a single-nucleotide *CIDEC* variant is associated with lipodystrophy, hypertriglyceridemia, and insulin resistance. To establish the unknown mechanistic link between CIDEC and maintenance of systemic glucose homeostasis, we generated transgenic mouse models expressing *CIDEC* (Ad-CIDECtg) and *CIDEC* E186X variant (Ad-CIDECmut) transgene specifically in the adipose tissue. We found that Ad-CIDECtg but not Ad-CIDECmut mice were protected against high-fat diet-induced glucose intolerance. Furthermore, we revealed the role of CIDEC in lipid metabolism using transcriptomics and lipidomics. Serum triglycerides, cholesterol, and low-density lipoproteins were lower in high-fat diet-fed Ad-CIDECtg mice compared to their littermate controls. Mechanistically, we demonstrated that CIDEC regulates the enzymatic activity of adipose triglyceride lipase *via* interacting with its activator, CGI-58, to reduce free fatty acid release and lipotoxicity. In addition, we confirmed that CIDEC is indeed a vital regulator of lipolysis in adipose tissue of obese humans, and treatment with recombinant CIDEC decreased triglyceride breakdown in visceral human adipose tissue. Our study unravels a central pathway whereby adipocyte-specific CIDEC plays a pivotal role in regulating adipose lipid metabolism and whole-body glucose homeostasis. In summary, our findings identify human CIDEC as a potential ‘drug’ or a ‘druggable’ target to reverse obesity-induced lipotoxicity and glucose intolerance.

Adipose tissue is a major regulator of metabolic health (1). Obesity is a significant health problem (1, 2), which broadly refers to excess adipose tissue. It is accompanied by increases in type 2 diabetes and cardiometabolic disease, the conditions linked to insulin resistance and glucose intolerance (1, 3). One of the main pathological causes is chronically elevated circulating free fatty acids (FFAs) and triglycerides (TGs) due to impaired adipose lipid homeostasis (1, 4). The identification of new therapeutic avenues aimed at maintaining lipid homeostasis and glucose tolerance is crucial to meet the need for the treatment of obesity and associated comorbidities.

Lipolysis of TGs stored in adipose tissue releases fatty acids (FAs) under conditions of metabolic need. Lipolysis of TGs to FFAs and glycerol requires three consecutive steps that involve three different enzymes: adipose triglyceride lipase (ATGL; also called desnutrin and PNPLA2), hormone-sensitive lipase (HSL), and monoacylglycerol lipase (MGL). ATGL is the rate-limiting enzyme for lipolysis in adipocytes and catalyzes the first step of hydrolysis of triglycerides to diacylglycerol (5, 6, 7, 8). CGI-58, a potent stimulator of ATGL catalytic activity, triggers ATGL-mediated lipolysis under physiologically stimulating conditions (9). In the basal state, perilipin A (PLIN1) prevents CGI-58 access to ATGL, restricting lipolysis (7, 9, 10, 11). Under basal conditions, ATGL is primarily localized in endoplasmic reticulum–related membranes in the cytoplasm and is in complex with its inhibitor, G0/G1 switch gene 2, G0S2 (12). Upon β-adrenergic stimulation, PKA activation results in PLIN1 phosphorylation at multiple sites, causing the release of CGI-58, which then binds and stimulates ATGL (10, 13). Translocation of unbound ATGL to lipid droplets, along with phosphorylated HSL, leads to acute activation of triglyceride hydrolysis. Prolonged β-adrenergic stimulation downregulates G0S2 and releases additional ATGL for sustained lipolysis.

Cell death–inducing DNA fragmentation factor-like effector (CIDE)-protein family members, CIDEA and CIDEC (also known as FSP27), play an important role in regulating fat metabolism in adipocytes (14, 15, 16, 17). CIDEC is an abundantly expressed lipid droplet–associated protein in adipocytes, where it regulates fat storage and release (14, 16). CIDEC expression is low in patients with insulin resistance and obesity (15), and weight loss surgery improves insulin sensitivity while increasing CIDEC expression (18). Further, a nonsense mutation (E186X) in CIDEC causes lipodystrophy, dyslipidemia, and insulin-resistant type 2 diabetes (19). *In vitro*, CIDEC appears to regulate ATGL-mediated lipolysis (16) or represses ATGL transcription (20). While these reports show a positive association between CIDEC and insulin sensitivity in humans, none showed a direct effect of CIDEC in maintaining systemic glucose homeostasis.

Although mouse and human CIDEC isoforms differ in their cellular and physiological effects, especially with regard to insulin signaling and glucose homeostasis (15, 16, 19, 21, 22, 23), all studies in humans, both *in vitro* and *in vivo*, unequivocally show a positive association of CIDEC with healthy metabolic phenotypes (15, 16, 18, 19, 24, 25, 26, 27, 28, 29, 30, 31). The present study establishes a direct link between human CIDEC and the maintenance of systemic lipid and glucose homeostasis. To investigate the adipose-specific role of human CIDEC and determine its mechanism of action, we generated two innovative transgenic mouse models expressing either full-length or mutant human CIDEC (E186X) in adipose tissue. We performed clinical human and experimental animal model investigations to identify a novel mechanism of CIDEC-mediated regulation of the lipolysis to protect against high-fat diet (HFD)–induced glucose intolerance.

## Results

### CIDEC expression negatively associates with increased lipolysis in visceral adipose tissue of patients with obesity

**Figure 1:**
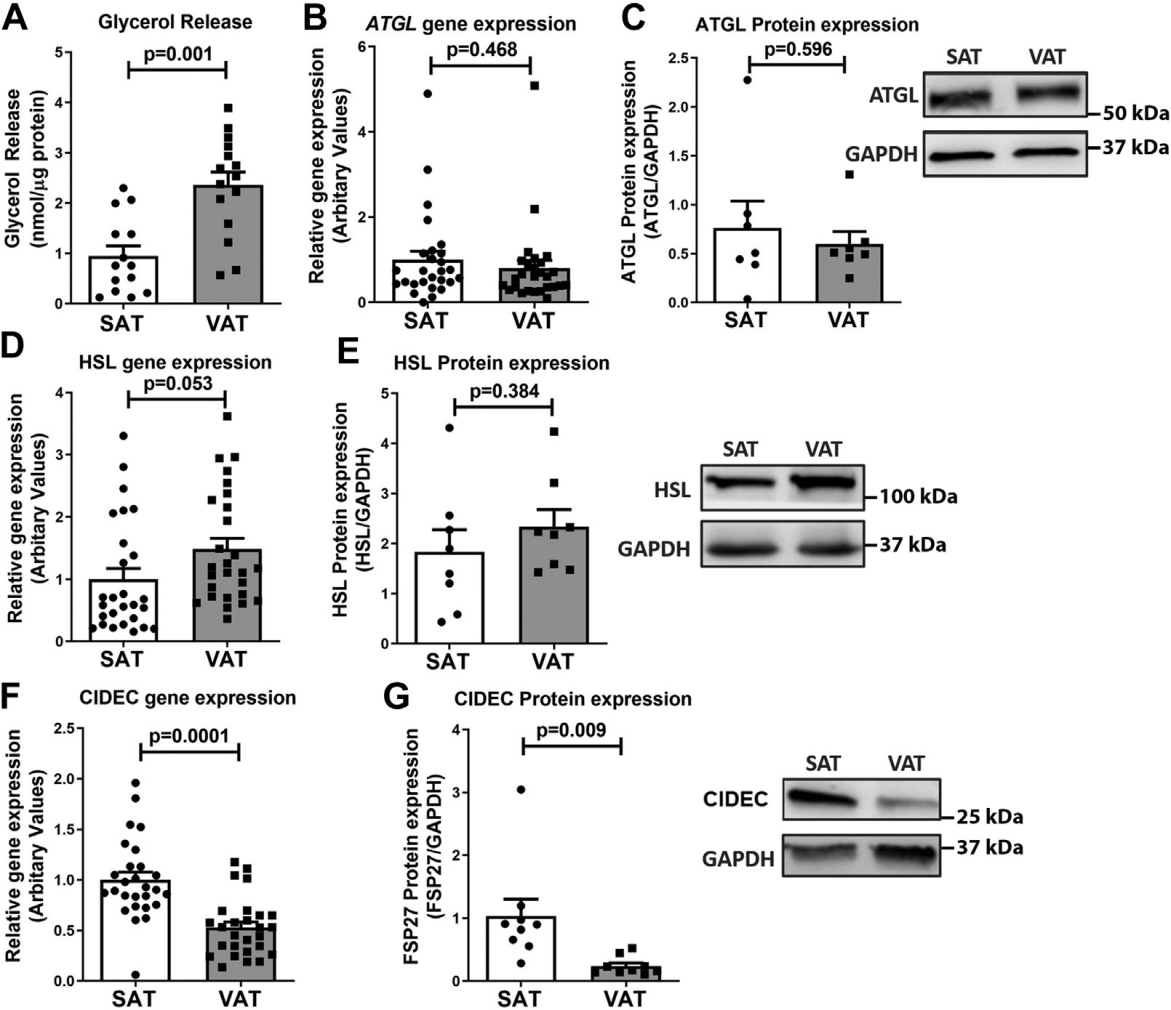
**CIDEC expression negatively associates with lipolysis in the adipose tissue of patients with obesity.***A*, basal lipolysis, assessed as glycerol release, was measured in 12 SAT and 15 VAT depots of human patients and normalized to total protein. Data are presented as mean ± SEM. *B*, basal *ATGL* mRNA in paired SAT and VAT depots of 27 human study participants. Data are presented as mean ± SEM. *C*, ATGL protein expression in SAT and VAT paired samples from eight randomly selected patients, with obesity among the 27 samples. Data are presented as mean ± SEM. *D*, basal *HSL* mRNA in paired SAT and VAT depots of 27 human participants. Data are presented as mean ± SEM. *E*, HSL protein expression in SAT and VAT paired samples from eight randomly selected patients with obesity. *F*, *CIDEC* mRNA in 27 paired SAT and VAT depots of patients with obesity. Data are presented as mean ± SEM. *G*, CIDEC protein level measured in paired SAT and VAT depots of eight randomly selected patients. Data are presented as mean ± SEM. For statistical significance, unpaired *t* test was used to analyze the significance between two groups. SAT, subcutaneous adipose tissue; VAT, visceral adipose tissue.

Obese human subjects undergoing planned bariatric surgery were recruited for the study; each provided paired subcutaneous adipose tissue (SAT) and visceral adipose tissue (VAT) samples at baseline, which were partitioned for different experiments. Table 1 shows the clinical characteristics of all participants. Glycerol release, a measure of lipolysis, was assessed in adipose tissue samples isolated during bariatric surgery. As expected (32), higher lipolysis was observed in VAT compared to SAT (Fig. 1*A*). Given this ∼2.5-fold difference, we measured the expression of lipases ATGL and HSL, which catalyze the breakdown of TGs. Unexpectedly, gene and protein expression of ATGL and HSL revealed no significant difference between VAT and SAT depots (Fig. 1, *B*–*E*). This was surprising because ATGL and HSL are major enzymes for lipolysis in adipocytes (33), with ATGL as the first and rate-limiting enzyme in TG hydrolysis (8, 9, 34). Because our previous studies showed that CIDEC regulates ATGL-mediated lipolysis in primary human adipocytes (16) and CIDEC positively correlates with reduced circulatory FFAs and insulin sensitivity in people with obesity (15, 30), we examined the expression of CIDEC in the paired VAT and SAT samples. Accumulation of visceral fat has been more closely and clinically linked to metabolic dysfunction, and we determined that CIDEC expression was significantly lower in VAT compared to SAT (Fig. 1, *F* and *G*).

**Table 1:** Clinical characteristics of obese human subjects

Parameter	Obese (n=52)
Age (years)	41 ± 14
Female (%)	87.3
BMI (kg/m^2^)	44.1 ± 7
Waist circumference (cm)	123 ± 7
Weight (kg)	120 ± 18
Insulin (mIU/ml)	17.1 ± 14
Glucose (mg/dl)	111 ± 49
HbA1C (%)	6.1 ± 1.9
HOMA-IR	6.5 ± 8.5
hsCRP (mg/dl)	9.0 ± 7.6
Triglycerides (mg/dl)	121 ± 60
Total cholesterol (mg/dl)	177 ± 48
HDL-C (mg/dl)	43.1 ± 10
LDL-C (mg/dl)	110 ± 37
Systolic BP (mmHg)	131.1 ± 14
Diastolic BP (mmHg)	74.5 ± 12
Diabetes mellitus (%)	35
Hypertension (%)	40
Hypercholesterolemia (%)	43

Data are mean ± SD.

BP, blood pressure.

### Generation and characterization of adipose-specific CIDEC transgenic mice

**Figure 2:**
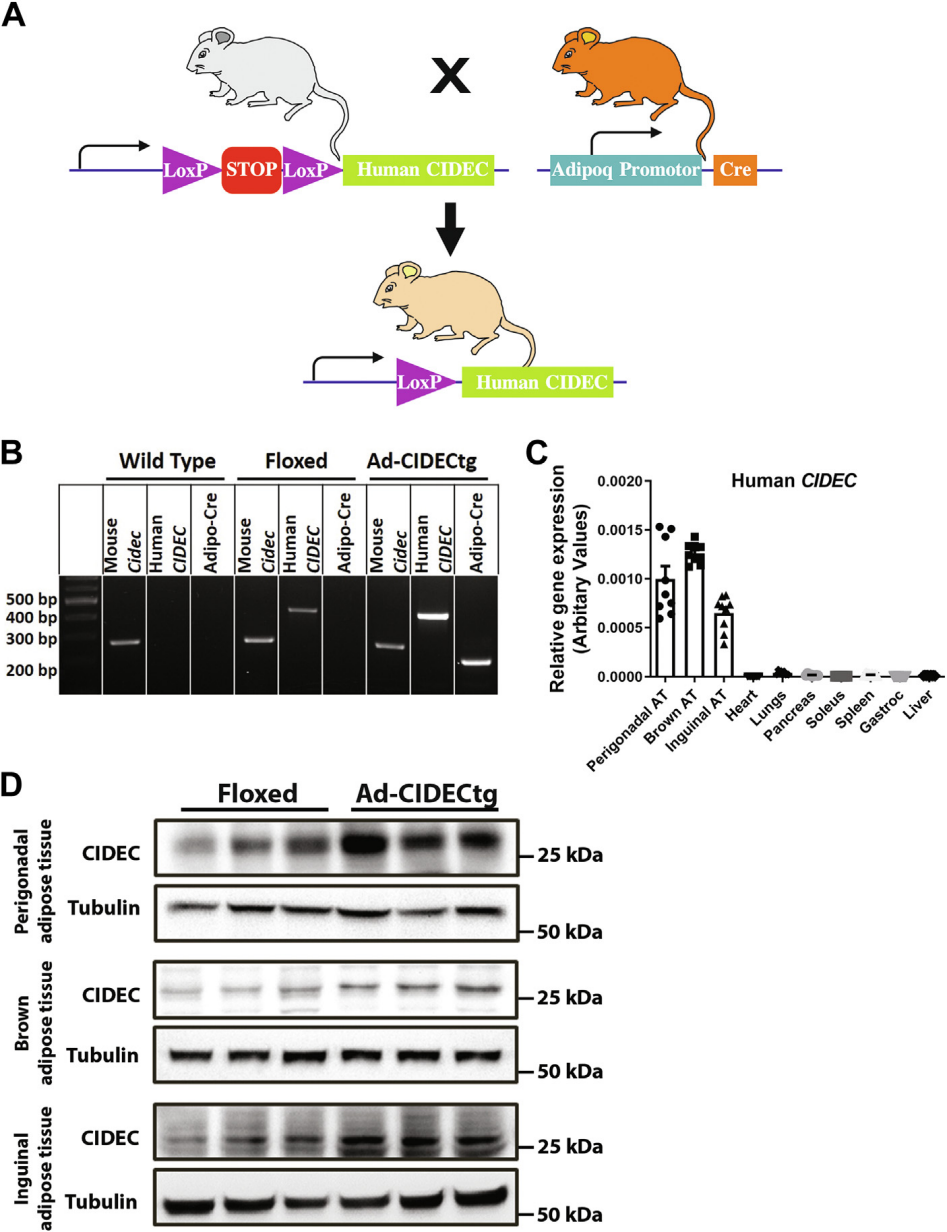
**Generation and characterization of Ad-CIDECtg transgenic mice expressing human CIDEC specifically in adipose tissue.***A*, schematic representation of the generation of Ad-CIDECtg mice expressing *CIDEC* in adipose tissue. *B*, genotyping of mice to assess the presence of human CIDEC and/or Cre recombinase. *C*, relative gene expression of human *CIDEC* in different organs of Ad-CIDECtg mice (n = 3 per group). *D*, protein expression of CIDEC in different adipose tissue depots including perigonadal *white* adipose tissue, inguinal adipose tissue (subcutaneous adipose tissue), and interscapular adipose tissue (*brown* adipose tissue) of male floxed and male Ad-CIDECtg mice (n = 3 per group).

To investigate the underlying molecular mechanism(s) of CIDEC-regulated adipose lipolysis and its effect on whole-body metabolism, we generated a transgenic mouse model expressing human *CIDEC* without altering endogenous mouse *Cidec* expression (Fig. 2*A*). To generate transgenic animals, a floxed stop codon followed by the human *CIDEC* expression cassette was inserted into the mouse *Rosa26* locus. To generate mice expressing human *CIDEC* in adipose tissue depots (Ad-CIDECtg), floxed mice were bred with B6.FVB-Tg (Adipoq-cre)1Evdr/J mice from Jackson Laboratory that express Cre recombinase enzyme under the control of mouse adiponectin promoter/enhancer regions (Fig. 2*A*). Specific primer pairs for human *CIDEC* and *Cre* recombinase were used to confirm the presence of either transgene (see Experimental procedures) (Fig. 2*B*). Mice expressing only human *CIDEC* were used as floxed controls, whereas mice expressing both human *CIDEC* and *Cre*, termed Ad-CIDECtg, were used as experimental models. To validate the adipose-specific expression of *CIDEC*, we performed quantitative RT-PCR and analyzed relative gene expression levels in various organs of Ad-CIDECtg mice. Quantitative RT-PCR results confirmed that *CIDEC* was expressed in adipose depots only (*i.e*., perigonadal, inguinal, and brown adipose depots) (Fig. 2*C*). CIDEC protein expression was assessed using Western blot analysis in insulin-sensitive organs. The antibody could not distinguish between human and mouse isoforms of CIDEC, so we expected increased expression of CIDEC in adipose tissue depots. Indeed, there was higher expression of total CIDEC protein (approximately 3-fold increase) in adipose depots (Fig. 2*D*).

### Metabolic characterization of Ad-CIDECtg mice

Ad-CIDECtg and floxed-littermate control mice were fed regular chow or HFD (60% kcal from fat) beginning at 3 months of age. Metabolic phenotyping was performed after 10 weeks of regular chow or HFD feeding. Body weight assessment indicated an almost equal increase in weight among transgenic and control mice (Fig. S1*A*). The slightly higher weight gain in Ad-CIDECtg mice was not statistically significant. White adipose tissue, brown adipose tissue, and liver collected after the completion of the metabolic study also did not significantly differ in weight between the same diet groups (Fig. S1*B*). Differences in food intake (Fig. S1*C*) and energy intake were not significant between floxed and Ad-CIDECtg mice fed a similar diet (Fig. S1*D*) nor was fat or lean mass normalized to body weight (Fig. S1, *E* and *F*). There was no significant difference in oxygen consumption, carbon dioxide exhalation, respiratory exchange ratio, energy expenditure, or ambulatory activity between Ad-CIDECtg mice and their littermate floxed controls (Fig. S1, *G* and *H* and S2).

### Ad-CIDECtg mice were protected against HFD-induced glucose intolerance

**Figure 3:**
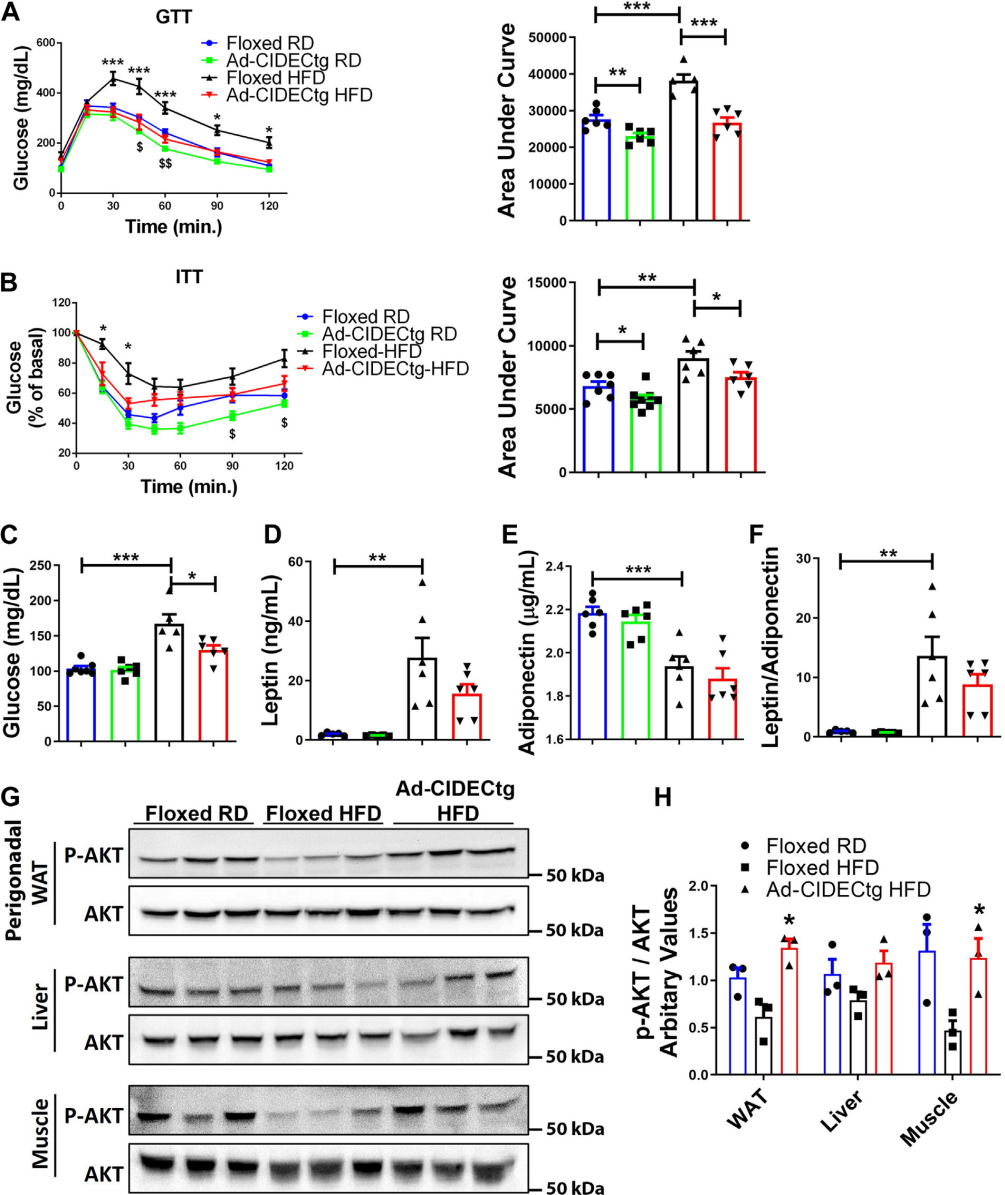
**Ad-CIDECtg mice exhibit protection against high fat diet (HFD)–induced glucose intolerance.***A*, i.p. GTT in 4.5 month old (3 months + 1.5 months HFD) male floxed or Ad-CIDECtg mice either on a regular diet (RD) or HFD for 10 weeks. The plot on the right-hand side represents area under the curve (AUC). ∗ signifies statistical significance between the HFD-fed groups, and ^$^ signifies statistical significance between the RD-fed groups. *B*, ITT of floxed or Ad-CIDECtg mice either on a RD or HFD. The plot on the right-hand side represents the AUC. ∗ signifies statistical significance between the HFD-fed groups, and ^$^ signifies statistical significance between the RD-fed groups. *C*, fasting blood glucose levels in mice. *D*, serum leptin levels, (*E*) serum adiponectin levels, and (*F*) leptin/adiponectin ratio. In *C*–*F*, *blue*, *green*, *black*, and *red* bars represent floxed RD, Ad-CIDECtg RD, floxed HFD, and Ad-CIDECtg HFD, respectively. *G*, Western blot analysis representing relative insulin-stimulated AKT phosphorylation (Ser473) levels in insulin-sensitive tissues including white adipose tissue, liver, and muscle (gastrocnemius), and (*H*) densitometry analysis of Western blots. Data are represented as mean ± SEM. For statistical analysis, two-way ANOVA followed by Bonferroni post hoc analysis was performed for (*A*) and (*B*). For other graphs, a student’s *t* test was applied to compare the significance between the two groups. ∗*p* < 0.05, ∗∗*p* < 0.01, ∗∗∗*p* < 0.001 and ^$^*p* < 0.05, ^$$^*p* < 0.01, ^$$$^*p* < 0.001 (n = 6–8 per group). GTT, glucose tolerance test.

We next evaluated the effect of HFD on glucose homeostasis in Ad-CIDECtg and their floxed-littermate controls. Ad-CIDECtg mice showed significantly higher glucose tolerance in an i.p. glucose tolerance test (GTT) compared to floxed-control mice fed HFD (Fig. 3*A*). Insulin (0.75 IU/kg) was injected i.p. in fasted mice. Consistent with GTT, Ad-CIDECtg mice exhibited improved insulin tolerance (Fig. 3*B*). Thus, Ad-CIDECtg mice were protected against HFD-induced glucose intolerance and insulin resistance. Measurement of fasting blood glucose revealed a significant reduction in glucose levels in Ad-CIDECtg mice fed HFD (Fig. 3*C*), indicating improved glucose homeostasis in Ad-CIDECtg mice. Serum leptin levels exhibited a decreasing trend, whereas serum adiponectin was unchanged in HFD-fed Ad-CIDECtg mice (Fig. 3, *D* and *E*). The leptin/adiponectin ratio, which positively correlates with type 2 diabetes (35), exhibited a decreasing trend in HFD-fed Ad-CIDECtg mice (Fig. 3*F*). Finally, to assess the effect of adipose tissue–specific expression of human CIDEC on organ-specific insulin sensitivity, mice were injected with insulin (i.p.) and measured AKT phosphorylation. AKT is a nodal protein in the insulin signaling pathway and a measure of insulin signaling in perigonadal adipose tissue, liver, and skeletal muscle. Ad-CIDECtg mice, when fed HFD, showed improved AKT phosphorylation in perigonadal adipose tissue and skeletal muscle, suggestive of improved systemic insulin sensitivity (Fig. 3, *G* and *H*). Also, we measured insulin levels before and after glucose injection in HFD-fed floxed-controls and Ad-CIDECtg mice. As expected, HFD-fed Ad-CIDECtg mice had slightly lower basal insulin levels compared to HFD-fed floxed-control mice. After the glucose injection, the control group showed higher levels of circulating insulin compared to transgenic mice at 2 min and 5 min time points. However, the significance could not be achieved due to the high level of variability in the insulin levels of floxed-controls compared to the Ad-CIDECtg mice. Subsequent time points showed comparable levels of circulating insulin in both groups. These data suggest that the pancreas is normal in both the groups and Ad-CIDECtg mice are more insulin sensitive when fed a HFD (Fig. S3). Overall, these results led us to conclude that adipose-specific expression of CIDEC regulates whole-body glucose homeostasis and insulin sensitivity.

### Transcriptome analysis of Ad-CIDECtg mice adipose tissue revealed improved lipid metabolism

**Figure 4:**
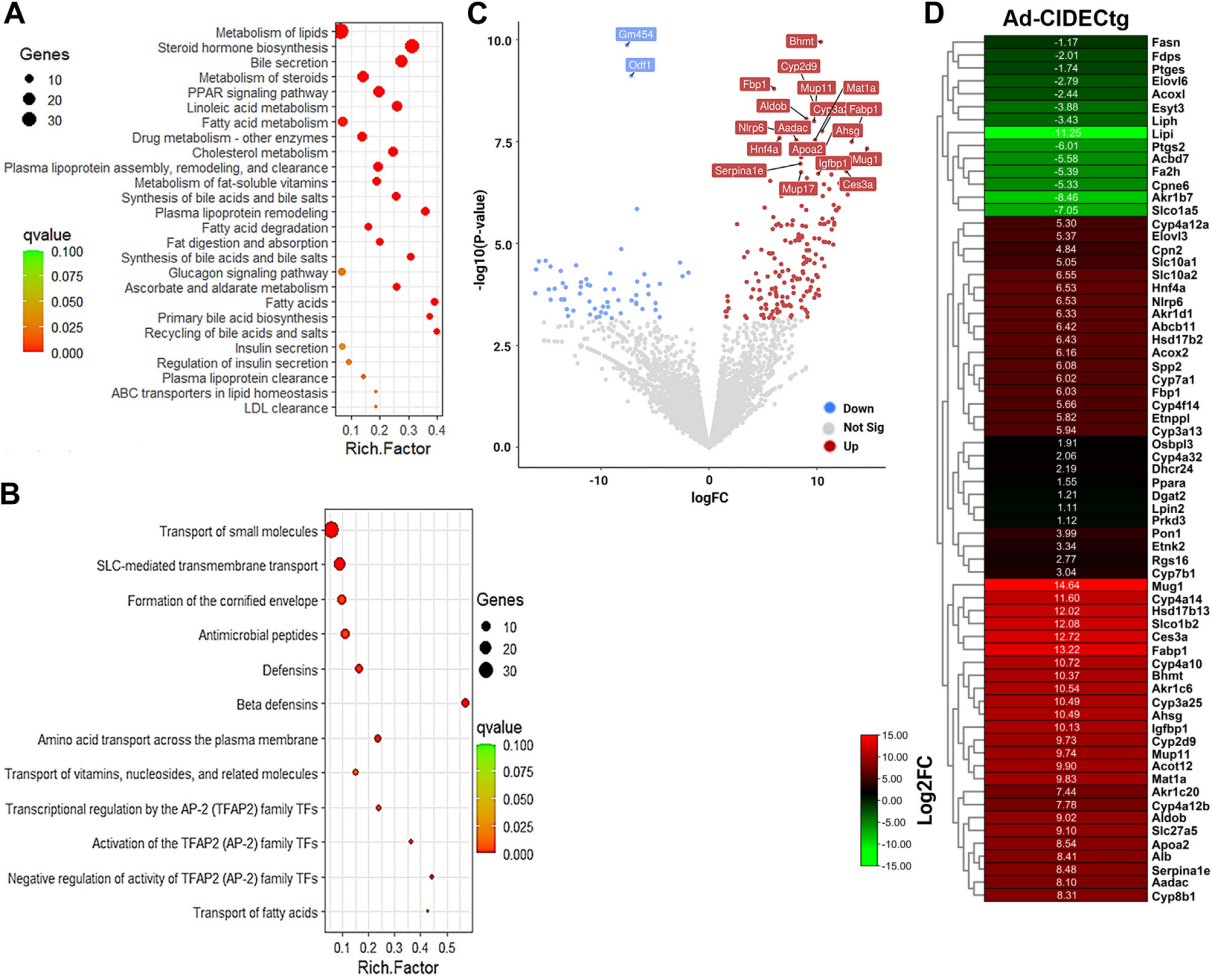
**RNA-seq analysis in white adipose tissue of floxed and Ad-CIDECtg mice.***A*, significantly enriched (q value <0.05) biological pathways among upregulated DEGs, and (*B*) downregulated pathways in adipose tissue of Ad-CIDECtg mice. The rich factor is the ratio of the number of DEGs to the total gene number in a pathway. Here, the q-value is a corrected *p*-value. The color and size of the dots represent the range of q-value and the number of DEGs mapped to the indicated pathways, respectively. *C*, volcano plot representing differently expressed genes in floxed *versus* Ad-CIDECtg mice. *D*, heat map showing altered genes associated with lipid metabolism in perigonadal adipose tissue of Ad-hCIDECtg mice. DEG, differentially expressed gene.

To identify changes in gene expression and metabolic pathways triggered by adipose tissue expression of human CIDEC in Ad-CIDECtg mice, we performed RNA-seq analysis in adipose tissue paired with pathway enrichment analysis. Differentially expressed genes (DEGs) between floxed and Ad-CIDECtg mice were identified using the EdgeR tool. After filtering by *p* < 0.05 (false discovery rate) and Log2FoldChange>1, we detected 1431 DEGs. Among these, 670 were downregulated and 761 were upregulated. Pathway analysis of these DEGs revealed 26 upregulated pathways (Fig. 4*A*) and 12 downregulated pathways (Fig. 4*B*). Interestingly, many upregulated genes were associated with lipid metabolism pathways. A volcano plot depicting upregulated (*red*), downregulated (*blue*), and unaltered (*gray*) DEGs is displayed in Figure 4*C*. Interestingly, most of the upregulated genes in adipose tissue of Ad-CIDECtg mice were associated with lipid homeostasis. The heat map in Figure 4*D* shows altered genes associated with lipid metabolism in Ad-CIDEC mice. Overall, our transcriptome analysis aligned with previous studies suggesting the role of CIDEC in lipid homeostasis (31).

### Serum lipid parameters and FFA species were reduced in Ad-CIDECtg mice

**Figure 5:**
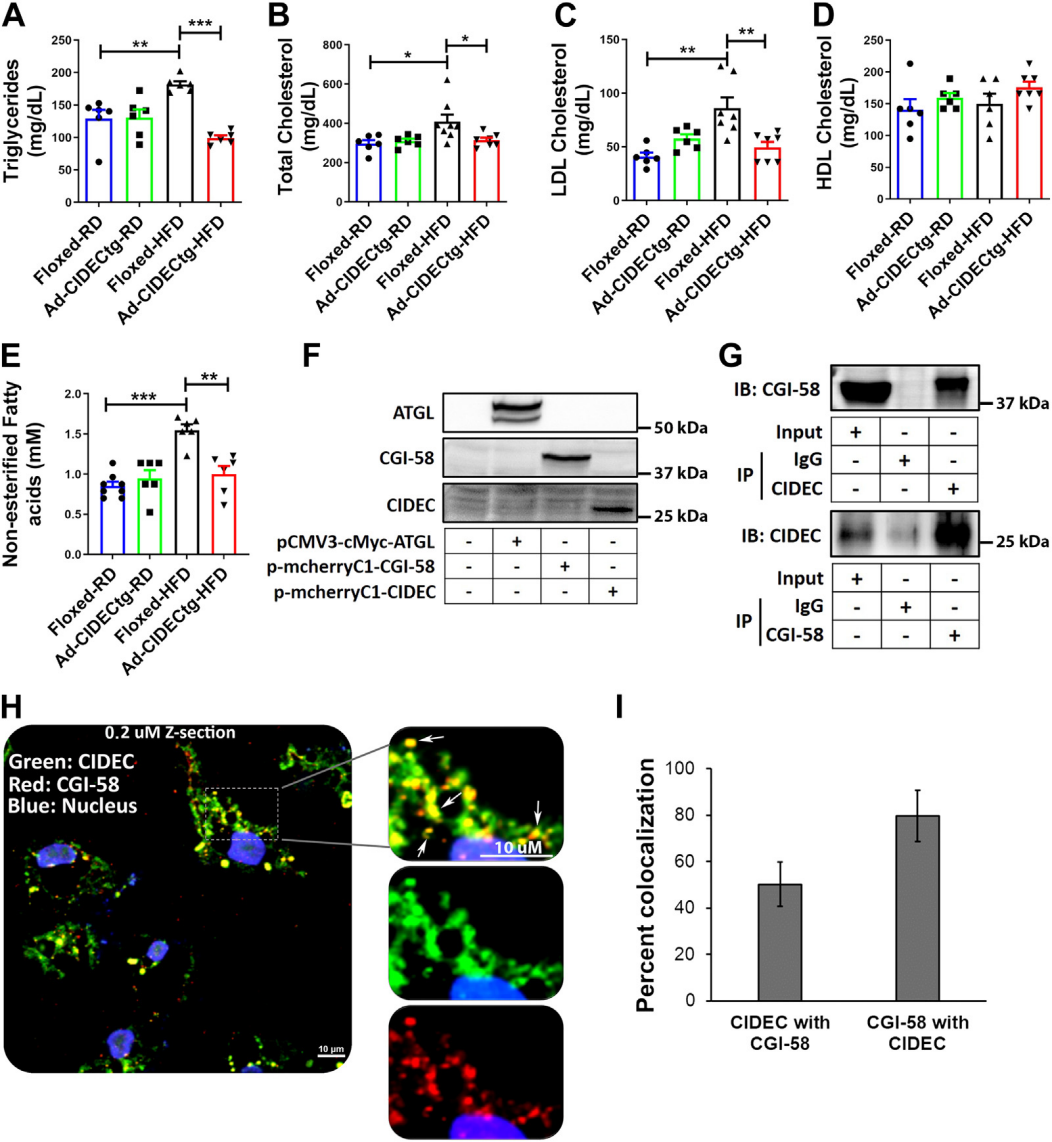
**CIDEC interacts with CGI-58.** Fasting serum lipid parameters were analyzed in floxed and Ad-CIDECtg mice fed regular chow or HFD. *A*, serum triglycerides, (*B*) serum total cholesterol, (*C*) low-density lipoprotein, (*D*) high-density lipoprotein, and (*E*) nonesterified fatty acids. *F*, Western blot analysis to confirm the overexpression of human ATGL, human CGI-58, and human CIDEC. *G*, immunoprecipitation of human CIDEC from cell lysates of human CIDEC and human CGI-58 overexpressing HEK-293 cells and immunoblotting against CGI-58 antibody (top panel). Immunoprecipitation of CGI-58 from cell lysates of human CIDEC and human CGI-58 overexpressing HEK-293 cells and immunoblotting against CIDEC (lower panel). *H*, a Z-slice of 0.2 μm showing localization of CIDEC (*green*) and CGI-58 (*red*) in culture human adipocytes. *Yellow* color shows colocalization of CIDEC and CGI-58. *I*, semiquantitative analysis of CIDEC colocalized to CGI-58 (*green* to *red*), and CGI-58 colocalized to CIDEC (*red* to *green*) (n = 10). HFD, high-fat diet.

We hypothesized that serum lipid parameters would be reduced in Ad-CIDECtg mice. Indeed, serum TG levels were significantly lower in HFD-fed Ad-CIDECtg mice (Fig. 5*A*). Total cholesterol (Fig. 5*B*) and low-density lipoprotein (LDL) levels (Fig. 5*C*) were also significantly reduced in HFD-fed Ad-CIDECtg mice. Levels of high-density lipoproteins (HDLs) in Ad-CIDECtg mice exhibited an increasing trend (Fig. 5*D*). Total nonesterified FAs were significantly lower in HFD-fed Ad-CIDECtg mice compared to littermate-floxed controls (Fig. 5*E*). To get a deeper insight into the FFA species, we performed lipidomics on serum samples from Ad-CIDECtg and floxed littermates, which showed a significant reduction in various FA species in serum FFA, cholesterol, and TG fractions in Ad-CIDECtg mice (Table S1). Next, we assessed adipocyte morphology and size in the perigonadal adipose tissue of floxed-controls and Ad-CIDECtg mice. Although the adipocyte size appeared slightly bigger in the Ad-CIDECtg mice, no significant change was observed in adipocyte number (Fig. S4). Overall, these results supported the role of CIDEC in improved lipid homeostasis.

### CIDEC interacts with CGI-58 and limits ATGL activation

Our previous *in vitro* study showed that CIDEC suppresses ATGL-mediated lipolysis in cultured adipocytes (16). However, the molecular action of CIDEC-mediated suppression of ATGL-mediated lipolysis is not yet known. No change in ATGL and HSL expression was observed in the adipose tissue of obese human subjects (Fig. 1); we therefore hypothesized that CIDEC might regulate FFA release *via* its action on ATGL catalytic activity. Since CGI-58 is a potent activator of ATGL, we tested whether CIDEC regulates ATGL activity *via* CGI-58. We used coimmunoprecipitation to investigate CIDEC and CGI-58 interaction. First, we overexpressed human-CGI-58, human-ATGL, and human-CIDEC in HEK293 cells and confirmed their expression by Western blot (Fig. 5*F*). Immunoprecipitation with CIDEC and immunoblotting against CGI-58 confirmed that CIDEC and CGI-58 interact (Fig. 5*G* top panel). Inversely, immunoprecipitation with CGI-58 and immunoblotting against CIDEC confirmed CGI-mediated pull down of CIDEC (Fig. 5*G* bottom panel). Next, we sought to determine the distribution of CIDEC and CGI-58 in cultured human adipocytes. Confocal microscopy on cells immunolabeled with CIDEC and CGI-58 antibodies revealed significant colocalization of CGI-58 and CIDEC (Fig. 5*H*). Semiquantitative measurement showed over 80% CGI-58 was colocalized with CIDEC in cultured human adipocytes (Fig. 5*I*).

**Figure 6:**
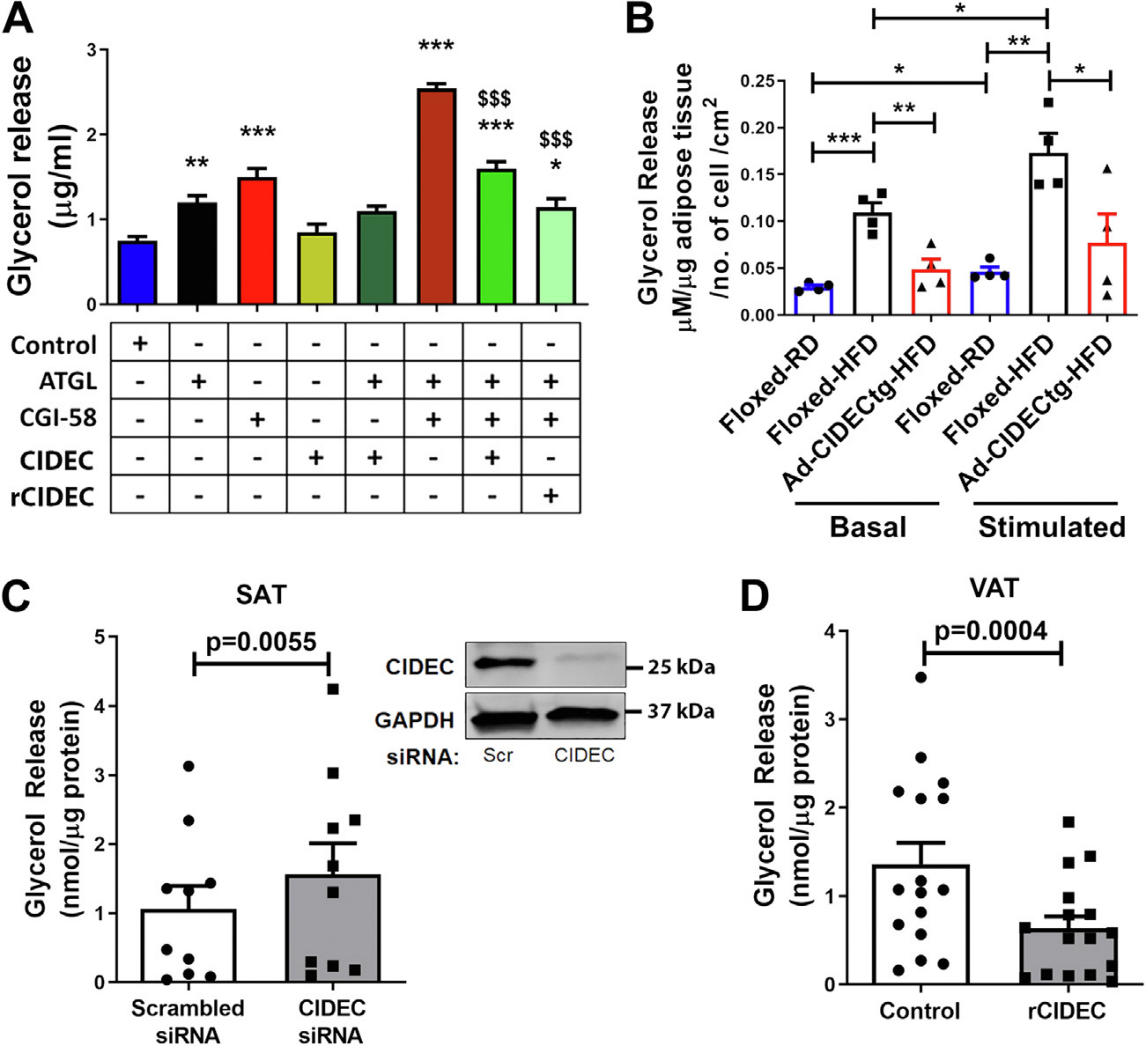
**CIDEC limits CGI-58-induced ATGL activation to release FFAs.***A*, measurement of glyceryl trilinoleate lipolysis upon addition of cell lysates overexpressing either ATGL, CGI-58, or CIDEC alone or in different combinations to assess ATGL-induced lipolytic activity. ∗ signifies statistical significance compared to controls, ^$^ signifies statistical significance compared to the lipolysis measured in column 6 (ATGL+CGI-58). One-way ANOVA was performed to analyze the significance between multiple groups (n = 3 per group). ∗*p* < 0.05, ∗∗*p* < 0.01, ∗∗∗*p* < 0.001 and ^$^*p* < 0.05, ^$$^*p* < 0.01, ^$$$^*p* < 0.001. *B*, glycerol release was assessed in *ex vivo* explants of white adipose tissue of mice either unstimulated or stimulation with CL-316243 (2 μM) for 2 h. Data are represented as mean ± SEM. For statistical analyses, a student’s *t* test was applied to compare two groups. ∗*p* < 0.05, ∗∗*p* < 0.01, ∗∗∗*p* < 0.001. *C*, glycerol release was assessed in the adipose tissue from SAT depot (n = 10) after siRNA-mediated *CIDEC* knockdown. Scrambled siRNA was used as a control. Top right panel is a representative Western blot showing siRNA-mediated CIDEC knockdown in SAT. *D*, glycerol release from the VAT depot (n = 16) after 24 h of control (DMSO) and recombinant CIDEC (rCIDEC) treatment. Data are presented as mean ± SEM. Paired *t* test was performed to analyze the significance between the two groups. DMSO, dimethyl sulfoxide; FFA, free fatty acid; SAT, subcutaneous adipose tissue; VAT, visceral adipose tissue.

To test our hypothesis that CGI-58-mediated stimulation of ATGL activity is impaired by CIDEC, ATGL catalytic activity was performed in the presence of ATGL, CGI-58, and CIDEC, either individually or in combination. ATGL or CGI-58 alone had a modest increase in lipolysis compared to controls; however, ATGL-mediated lipolysis was significantly enhanced by CGI-58 (Fig. 6*A*). Interestingly, adding CIDEC did not affect lipolysis in the presence of ATGL alone, but it significantly reduced lipolysis induced by the combination of ATGL and CGI-58 (Fig. 6*A*), indicating that CGI-58 action on ATGL was inhibited by CIDEC. To further confirm the effect of CIDEC on CGI-58 action, we used recombinant CIDEC protein (rCIDEC) and performed a lipase activity assay. rCIDEC significantly reduced the effect of CGI-58 on the catalytic activity of ATGL (Fig. 6*A*).

To confirm the role of human CIDEC transgene in adipose tissue lipolysis *in vivo*, we measured basal and stimulated lipolysis in isolated mouse white adipose tissue from Ad-CIDECtg mice and the littermate controls. Adipose tissue explants were incubated with or without CL-316,243 (2 μM) for 2 h, and glycerol release was measured. Under both basal and stimulated conditions, adipose tissue from Ad-CIDECtg mice showed significantly reduced glycerol release (Fig. 6*B*), thus confirming the role of adipose-specific expression of CIDEC in decreasing lipolysis. Furthermore, to assess the role of CIDEC in lipolysis in human adipose tissue, we used loss- and gain-of-function experiments using CIDEC siRNA and rCIDEC, to deplete or overexpress CIDEC, respectively, in human adipose tissue explants. Interestingly, siRNA-mediated CIDEC depletion in SAT increased lipolysis (Fig. 6*C*), whereas treatment of human VAT with rCIDEC significantly decreased lipolysis (Fig. 6*D*).

### Transgenic mice expressing mutant human-CIDEC were not protected from HFD-induced insulin resistance

**Figure 7:**
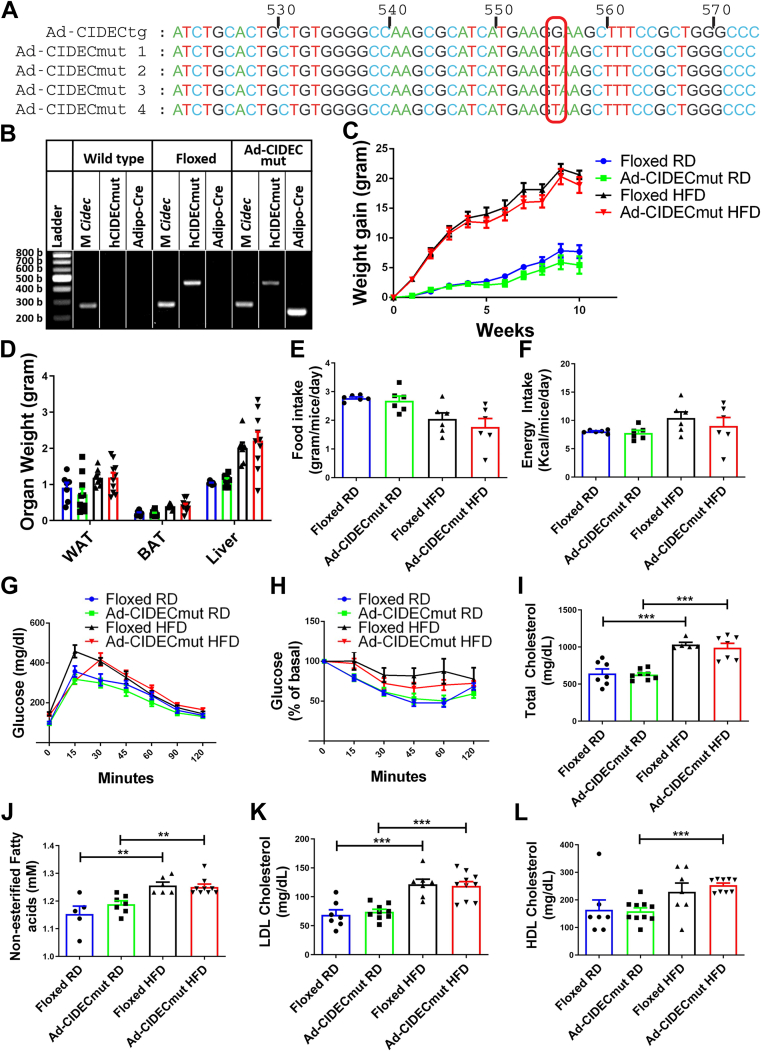
**Generation, characterization, and metabolic studies in Ad-CIDECmut mice.***A*, DNA sequence of human *CIDEC* transgene expressed in Ad-CIDECtg mice compared to mutant *CIDEC* transgene expressed in Ad-CIDECmut mice. *B*, genotyping of Ad-CIDECmut mice for the presence of mutant *CIDEC* and/or *Cre* recombinase. M *Cidec* represents mouse Cidec gene. *C*, body weight gain of floxed and Ad-CIDECmut mice fed either regular chow or HFD. *D*, organ weight of floxed and Ad-CIDECmut mice. *Blue*, *green*, *black*, and *red* bars represent floxed RD, Ad-CIDECmut RD, floxed HFD, and Ad-CIDECmut HFD, respectively. *E*, food intake and (*F*) energy intake of floxed and Ad-CIDECmut mice fed either regular chow or HFD. *G*, GTT and (*H*) ITT of mice. *I–L*, fasting serum total cholesterol (*I*), nonesterified fatty acids (*J*), LDL cholesterol (*K*), and HDL cholesterol (*L*) of floxed and Ad-CIDECmut mice fed either regular chow or HFD. Data are represented as mean ± SEM. For statistical analysis, two-way ANOVA followed by Bonferroni post hoc analysis was performed for (*G*) and (*H*). For other graphs, a student’s *t* test was applied to compare the significance between the two groups. ∗*p* < 0.05, ∗∗*p* < 0.01, ∗∗∗*p* < 0.001 (n=6–8 per group). GTT, glucose tolerance test; HFD, high-fat diet; ITT, insulin tolerance test.

Finally, to confirm the consequential role of human CIDEC in FA homeostasis and protection against HFD-induced insulin resistance, we generated an innovative adipose tissue-specific mouse model expressing human *CIDEC* (556G→T) mutation, Ad-CIDECmut. A known single nucleotide polymorphism in *CIDEC* produces a nonfunctional protein causing partial lipodystrophy, hypertriglyceridemia, and insulin-resistant type 2 diabetes (19). The transversion mutation of guanine to thymine at complementary DNA (cDNA) nucleotide position 556 in exon 6 of *CIDEC* results in a premature stop codon (19). Since CIDEC was suggested to undergo homodimeric or heterodimeric functional interactions (36, 37), we hypothesized that a mouse model expressing the *CIDEC* (556G→T) variant might show a dominant-negative effect of CIDEC on metabolic parameters. The floxed Ad-CIDECmut mouse model was generated in the same manner as the human CIDEC full-length in Figure 2. Sequencing results confirmed the presence of this mutation in Ad-CIDECmut mice (Fig. 7*A*). Crossing Ad-CIDECmut floxed mice with mice expressing Cre recombinase under adiponectin promoter expressed the *CIDEC* mutant variant in the adipose tissue (Fig. 7*B*). Ad-CIDECmut and floxed-littermate control mice were fed either regular chow or HFD for 10 weeks beginning at 3 months of age. No change in weight gain was observed between the regular chow or HFD groups (Fig. 7*C*). Weight of perigonadal adipose tissue, brown adipose tissue, and liver was unaltered between groups (Fig. 7*D*). Similarly, no difference was observed in food or energy intake (Fig. 7, *E* and *F*). GTT and insulin tolerance test (ITT) analysis revealed no significant differences (Fig. 7, *G* and *H*), implying that the expression of the *CIDEC* mutant transgene in mouse adipose tissue does not induce any metabolic alterations. Indirect calorimetry to analyze consumed oxygen, exhaled carbon dioxide, respiratory exchange ratio, and ambulatory activity revealed no significant difference between groups (Fig. S5). Finally, we assessed fasting serum lipids including total cholesterol, nonesterified FAs, LDL cholesterol, and HDL cholesterol. No significant differences were observed among lipid parameters between Ad-CIDECmut and floxed-controls fed regular chow or HFD (Fig. 7, *I*–*L*). In addition, there was no observable difference in size or morphology of adipocytes from either chow diet-fed or HFD-fed floxed *versus* Ad-CIDECmut mice (Fig. S6). Overall, the results revealed that the expression of mutant *CIDEC* transgene in adipose tissue of mice does not exhibit any physiological or metabolic effects. Thus, Ad-CIDECmut mouse model acted as a negative control displaying *CIDEC* (556G→T) as nonfunctional.

## Discussion

Here, we provide evidence for a direct role of human CIDEC in regulating systemic lipid and glucose homeostasis. CIDEC is a key protein in maintaining lipid droplet dynamics and lipolysis in adipocytes (16, 38, 39), and there is a uniformly positive correlation between CIDEC expression and insulin sensitivity in humans (15, 18, 19, 25, 30, 31). Interestingly, a single nucleotide mutation in *CIDEC* that causes premature truncation is associated with profound metabolic dysfunction (19). We sought to establish the mechanistic role of human CIDEC in lipid metabolism and whole-body glucose homeostasis. This investigation was spurred by our clinical observations that CIDEC plays a key role in insulin sensitivity regulation and lipolysis in human adipose tissues that are not fully explained by ATGL and HSL expression levels. We generated two mouse models expressing a single copy of full-length human *CIDEC* (Ad-CIDECtg) or the *CIDEC* truncation variant (Ad-CIDECmut) in adipose tissue. Ad-CIDECtg, but not Ad-CIDECmut mice, exhibited reduced fasting glucose levels compared to their floxed-littermate controls. Similarly, Ad-CIDECtg mice were protected against HFD-induced insulin resistance and exhibited lower circulating FFA. Our RNA-seq analysis revealed changes in lipid metabolism pathways in the adipose tissue of Ad-CIDECtg mice. Lipidomics of serum samples confirmed a decrease in every measured serum FA species in HFD-fed Ad-CIDECtg mice. Together, our findings demonstrate that CIDEC plays a key role in controlling lipolysis and whole-body glucose homeostasis, and mechanistic studies revealed that CIDEC regulates the catalytic activity of ATGL to restrict the release of FFAs by interacting with CGI-58.

Obesity-induced downregulation of CIDEC is more pronounced in VAT than SAT, and weight loss surgery restores CIDEC expression levels and improves insulin sensitivity in humans (18). Indeed, our clinical data showed that obese subjects had lower CIDEC expression in VAT than SAT. Surprisingly, patients with obesity displayed significantly increased visceral adipose lipolysis without any differential expression in ATGL and HSL. However, our study did not exclude the possibility of changes in HSL phosphorylation, which is required for HSL activity (40). The direct effect of CIDEC on lipolysis was evident when we modulated CIDEC expression in human adipose tissue. Recombinant CIDEC reversed lipolysis and restored insulin sensitivity in human VAT (30). These results confirmed the negative association of CIDEC expression with lipolysis and insulin resistance in human adipose tissue (15, 19, 27, 30).

Gene orthologs often, but not always, have a similar function in closely related species (41). Similarly, whole-body CIDEC deletion in mice and CIDEC mutation in humans lead to dramatically different phenotypes (19, 21, 23). Interestingly, CIDEC KO mice exhibit higher mitochondrial oxidation and insulin sensitivity (21, 23), but adipose tissue-specific knockdown causes hepatosteatosis and insulin resistance in HFD-fed mice (42). For this reason, we kept the endogenous mouse CIDEC intact in our transgenic mice models. We sought to determine the functional role of human CIDEC in whole body metabolism and elucidate the mechanism of action. A similar human transgene expressed in mice was used to elucidate the physiological role of the human *CIDEA* gene (43). This study showed that transgenic mice expressing human *CIDEA* in adipose tissue display improved metabolic profile through expansion of adipose tissue, complementing studies in humans (44, 45, 46).

Optimal ATGL activity must be maintained to provide FAs to tissues as a source of energy. ATGL is a rate-limiting enzyme during lipolysis (7). Yet, its role extends beyond adipose tissue: ATGL null mice die prematurely due to the accumulation of excess lipids in cardiomyocytes (47). However, these mice exhibited improved glucose homeostasis, which could be explained by an accumulation of lipids in adipose tissue due to restricted lipolysis, which in turn reduces circulating FFAs and accentuates the dependency of other tissue on glucose homeostasis, leading to glucose tolerance and insulin sensitivity (47). A slight increase in adipocyte size along with insulin sensitivity in our Ad-CIDECtg mice could mainly be attributed to the reduced circulating FFAs. Although, increase in adipocyte size has been positively correlated with insulin resistance, there is no direct evidence suggesting that adipocyte hypertrophy could alone diminish insulin responsiveness. Previous findings that large adipocytes do not directly correlate with insulin resistance support our results (48, 49, 50). Adipose-specific deletion of ATGL resulted in a similar phenotype exhibiting a marked increase in glucose and insulin sensitivity (51). Our evidence of CIDEC involvement in regulating lipid homeostasis led us to further investigate the intricate lipolytic cascade to determine the place of CIDEC in the molecular pathway. We unveiled a previously unknown role of CIDEC in inhibiting CGI-58-mediated activation of ATGL. Our results showed that CIDEC interacts with CGI-58 to inhibit its action on ATGL activity. A limitation of our study is that it could not show co-immunoprecipitation of endogenous CIDEC and CGI-58 using adipocyte samples. Perhaps the high detergent concentration used to isolate the lipid-droplet associated proteins disrupts their interaction. Our analysis of high-resolution confocal 0.2 μm thin Z-sections clearly showed the close proximity of CIDEC and CGI-58, and the coimmunoprecipitation using HEK cells showed their interaction. It is yet not clear if the CIDEC and CGI-58 binding is direct or indirect. The consequences of functional interaction of these proteins were confirmed by the ATGL activity assay where CIDEC inhibited the action of CGI-58 on ATGL-mediated lipolysis. Further studies are required to identify the role of CIDEC in exercise and fasting-mediated increase in lipolysis because both physiological conditions have common pathophysiology of increased lipolysis required to maintain energy levels in the body *via* the supply of FFAs.

We hypothesized that the expression of the human 556G→T variant in the adipose tissue of mouse would have a dominant negative effect on lipolysis and insulin resistance since CIDEC undergoes homodimeric or heterodimeric functional interactions (36). However, there was no difference in the metabolic phenotype of floxed-controls and Ad-CIDECmut mice, showing that the mutation (556G→T) rendered CIDEC nonfunctional. This truncation is in the C terminus of CIDEC, which we earlier identified to be the most active region in regulating lipid droplet dynamics (38), TG breakdown, and in protecting adipocytes against FFA-mediated insulin resistance (16, 38). Ad-CIDECmut mice did not show protection from increased serum lipids or impaired glucose and insulin tolerance, further confirming the physiological role of full-length CIDEC in regulating glucose and lipid homeostasis.

In conclusion, our findings support a novel mechanism by which CIDEC maintains adipose tissue homeostasis by regulating the lipolytic activity of the rate-limiting enzyme ATGL, thus reducing HFD-induced lipotoxicity and glucose intolerance. This mechanism also explains why a decrease in CIDEC, as observed in the obese state or patients with *CIDEC* SNPs, could result in FFA flux, leading to insulin resistance and related metabolic derangements. Indeed, *ex vivo* recombinant CIDEC decreased FFA release and restored insulin signaling in adipose tissue of obese humans (30). Furthermore, our recent study showed that FSP27-knockout (Cidec-knockout) mice presumed to be metabolically healthy based on glucose utilization and oxidative metabolism are unhealthy in terms of exercise capacity and muscular performance (52). Overall, these studies suggest CIDEC as a potential ‘drug’ or a ‘druggable’ target to reverse obesity-induced lipotoxicity and glucose intolerance.

## Experimental procedures

### Selection criteria of human subjects

Consecutive subjects, both men and women, with longstanding obesity (body mass index ≥35 kg/m2, age ≥18 years), enrolled in the Boston Medical Center Bariatric Surgery Program, were recruited into the study. Subjects with unstable medical conditions or pregnancy were not eligible for bariatric surgery and thus excluded. The study was approved by the Boston University Medical Center Institutional Review Board. Written consent was obtained from all participants.

### Collection of human tissue

Adipose tissue samples were collected intraoperatively from two separate regions. Samples of subcutaneous and visceral adipose tissue were collected intraoperatively from the lower abdominal wall and greater omentum, respectively, during planned bariatric surgery, as previously described (53, 54). Each subject provided one biopsy specimen from the subcutaneous and visceral fat depot. All biopsies were performed under fasting conditions.

### Western immunoblot analysis of human samples

Protein was extracted from adipose tissue by homogenization. Ice-cold 1X lysis buffer (Cell Signaling) supplemented with protease inhibitor cocktail and phosphatase inhibitor II and III (Sigma–Aldrich). All samples were assayed for protein content using Bradford's method. Thirty-five micrograms of total protein was subjected to electrophoresis in SDS-polyacrylamide gel under reducing conditions and blotted to a nitrocellulose membrane using the Bio-Rad Transblot Turbo Transfer system. The membranes were blocked in Tris-buffered saline (TBS) with 0.1% Tween-20 and 5% bovine serum albumin (BSA) for 1 h at room temperature (RT) and then incubated overnight with primary antihuman antibodies against respective proteins (1:500–1000 dilution) at 4 °C. Membranes were then washed off using TBS and incubated with horseradish peroxidase (HRP)–conjugated secondary anti-rabbit IgG (R&D System) for 1 h at RT. Immune complexes were detected with the enhanced chemiluminescence ECL detection system (Bio-Rad). Densitometric analysis of bands was performed using the ImageQuant LAS 4000 biomolecular imaging system (GE Healthcare). GAPDH was used as a loading control to normalize the quantification.

### Lipolysis assessment of human adipose tissue samples

The subcutaneous and visceral fat (0.5–1 g) were cut into 1 to 2 cm explants, washed, and incubated in Phenol-Red free Dulbecco's modified Eagle's medium (DMEM) with 2% FA-free BSA for 2 h at 37 °C at basal conditions. At the end of the experiment, media was collected, and glycerol content in the media was measured colorimetrically at 540 nm using the Glycerol Cell-Based Assay Kit (Cayman Chemical) against a set of glycerol standards. Tissue from each experiment was collected and homogenized to measure total protein content as described in the Western immunoblot analysis before. Data were calculated as nanomole of glycerol.

### Gene expression in human samples

Fragments of subcutaneous and visceral human adipose tissue were collected in RNAlater solution (Sigma–Aldrich) and stored at −80 °C. Samples were homogenized, and total RNA was isolated using RNeasy Mini kits (Qiagen). The RNA (0.5–1 μg) was retrotranscribed with high-capacity cDNA Synthesis Kits (Applied Biosystems). Quantitative real-time PCR reactions were performed using TaqMan gene expression assays in a ViiA7 PCR system (Applied Biosystems). Results were analyzed with the ΔΔCt method using GAPDH as a reference.

### Generation of Ad-CIDECtg mice

Transgenic mice lines were generated using Rosa26 knock-in system. The human CIDEC gene was cloned in ROSA26-CMV-loxSTOPlox vector, and mice were generated according to the standard approach by ES cells (55). Generated mice were bred with WT C57BL/6 mice for the next (six) generations and monitored for the presence of human CIDEC transgene using standard genotyping method with human CIDEC-specific primer pairs as described later. The transgenic-floxed mice positive for human CIDEC transgene were selected and crossed with the mice containing Cre-recombinase under adiponectin promotor (B6.FVB-Tg (Adipoq-cre)1Evdr/J mice purchased from Jackson Laboratory) to express human CIDEC specifically in adipocytes (Ad-CIDECtg mice). The mice were maintained on a regular chow diet for 3 months. At 3 months of age, both floxed-controls and Ad-CIDECtg mice were shifted to either a standard chow diet (10% calories from fat) or a HFD (60% calories from fat) for the next 10 weeks. Body weight was monitored weekly. At the end of the experiment, mice were fasted overnight, either pulsed or not pulsed with insulin (0.75 IU/kg of body weight), and blood and organs were collected. Serum was isolated from the blood by centrifugation, and organs were processed as per the specific analysis. IACUC approved all the protocols at the OHIO University, Athens, OH.

### Genomic DNA isolation and genotyping

Genomic DNA was isolated from the mice's ear punch using the NaOH method. Briefly, ear samples were collected in 1.5 ml microcentrifuge tube, to which 50 μl of NaOH-EDTA solution (25 mM NaOH, 0.2 mM EDTA) was added. The microcentrifuge tube was then heated at 100 °C for 1 h. Samples were centrifuged briefly, and 50 μl Tris–HCl solution (40 mM Tris–HCl, pH 5.0) was added. Samples were vortexed briefly and used for amplification of specific sequences.

### GTT and ITT

For the analysis of glucose homeostasis, the GTT and the ITT were performed. For GTT, mice were fasted overnight. After fasting, 1.5 g/kg of glucose was injected intraperitoneally. Blood glucose was monitored at different time points from 0 to 120 min using GE100 blood glucose monitoring system glucometer (GE Healthcare). For ITT, mice were fasted for 6 h. After fasting, 0.75 IU/kg insulin (Sigma–Aldrich) was then injected intraperitoneally. Blood glucose was monitored at different time points from 0 to 120 min. GE100 blood glucose monitoring system glucometer (GE Healthcare) was used to measure blood glucose concentrations.

### Body composition analysis

For the analysis of body fat distribution, mice were placed in a whole body composition analyzer (Bruker minispec live mice analyzer, LF50). Whole body fat mass and whole body lean mass data were collected and normalized with the total body weight of mice.

### Metabolic profiling of mice

To assess the changes in metabolic rate and energy expenditure, mice were placed in the Comprehensive Lab Animal Monitoring System (CLAMS) (Columbus Instruments). Mice were acclimatized for the initial 2 days in CLAMS. The changes in the rate of VO2, VCO2, food intake, and movements were recorded for the next 2 days. Mice were then fasted overnight to assess changes in the metabolic parameters during fasting. Data were recorded and was analyzed using the CalR web tool (56).

### Glycerol release estimation

Adipose tissue from animals was freshly isolated. Small tissue explants were washed three times with PBS and kept in DMEM media supplemented with 10% fetal bovine serum at 37 ^°^C for 2 h. Tissue samples were then either treated with vehicle (water) or were stimulated with 2 μM of CL-316243 for the next 2 h to induce lipolysis in a serum-free medium. Subsequently, media were collected, and glycerol was estimated using free glycerol reagent (Sigma–Aldrich, catalog no.: F6428).

### RNA isolation and quantitative real-time PCR analysis

Quantitative real-time PCR analysis was performed to assess relative gene expression as described earlier (57). For RNA extraction, tissue samples stored at −80 °C in TRIzol (Invitrogen) were thawed and homogenized using a bead beater. RNA was isolated using the manufacturer’s instructions. Five hundred nanograms of RNA was used to prepare cDNA using the RevertAid first-strand cDNA synthesis kit (Thermo Fisher). Quantitative real-time PCR was performed using the cDNA, gene-specific primer pairs, and SYBR Green master mix (Thermo Scientific).

### Western blotting

As described previously (58), adipose tissue was homogenized using bead beater in mammalian cell lysis buffer (G Biosciences) supplemented with protease and phosphatase inhibitors. Samples were centrifuged twice to isolate the clear protein fraction. An equal amount of protein (μg) was used to prepare SDS-PAGE samples. After adding the loading dye, samples were resolved on SDS-PAGE and subsequently transferred to the polyvinylidene difluoride membrane. The membrane was then blocked and probed with primary overnight followed by washing three times with TBS with Tween-20 buffer and secondary antibodies (diluted in 4% BSA in TBS with Tween-20) for the next 2 hrs. Finally, the blots were developed using HRP substrate (Immobilon Western Chemiluminescent HRP substrate, Millipore) and visualized under chemiluminescence, and images were captured using BioRad Imager.

### Leptin and adiponectin ELISA

Leptin (DY498) and adiponectin (DY1119) ELISA kits from R&D Biosystems were used to measure serum concentrations of leptin and adiponectin, respectively, following manufacturer’s instructions. As discussed previously (59), a 96-well plate was overnight coated with capture antibody 100 μl/well of 2 μg/ml concentration. The wells were washed with PBS with Tween-20 (PBST) and then blocked with 1% BSA in PBST for the next 1 h. Again, wells were washed with PBST, and 100 μl of the sample (for leptin, serum was diluted 10 times, and for adiponectin, serum was diluted 100 times) was added in each well along with the standard. Post 2 h incubation, wells were washed with PBST, and 100 μl of detection antibody was added to the wells (for leptin 200 ng/ml and adiponectin 25 ng/ml). After 2 h, wells were washed, and 100 μl of Streptavidin-HRP was added for 20 min. Wells were washed with PBST, and 100 μl of substrate solution was added for the next 20 min followed by the addition of 50 μl of stop solution (2N H_2_SO_4_). The absorbance was determined using a spectrophotometer at 450 nm.

### Total cholesterol quantification

Total cholesterol was assessed in serum samples of fasted mice using cholesterol kit from Randox laboratories (CH200) as per the manufacturer’s protocol. Briefly, 5 μl of serum or standard was taken in a 96-well plate. One-hundred fifty microliters of Reagent I was added to each well keeping one well for blank control. The reaction mixture was incubated at 37 °C for 5 min followed by measurement of absorbance at 500 nm. Data were collected and absorbance values were converted to actual concentration by plotting against a standard curve.

### Assessment of serum TGs

As described earlier (60), serum TGs were estimated using a TG estimation kit (TR210) from Randox laboratories as per the manufacturer’s protocol. Briefly, 5 μl of serum sample or standard was taken in a 96-well plate. Two-hundred microliters of Reagent R1 was added and the reaction was incubated at 37 °C for 10 min keeping one well as blank control. Postincubation absorbance was measured by spectrophotometer at 500 nm. Data were collected and absorbance values were converted to actual concentration by plotting against a standard curve.

### HDL and LDL estimation

HDL and LDL were estimated using kits (CH8311, CH8032) purchased from Randox laboratories as per manufacturer’s protocol. Briefly, 3 μl of serum sample or standard was used in a 96-well plate. About 90 μl of Reagent 1 was added to each well and the reaction was incubated at 37 °C for 5 min. It was followed by the addition of 30 μl of Reagent 2. The absorbance was read at 600 nm first after 30 s (A1) and then after 150 s for HDL and 300 s for LDL (A2). Actual absorbance was calculated by A2-A1. Absolute values were obtained by plotting against a standard curve.

### Adipose tissue imaging

Explants of fresh adipose tissue were washed with PBS and fixed in 1 ml of 4% paraformaldehyde in PBS for 1 h at RT. The explants were then washed thrice with PBS, followed by staining with wheat germ agglutinin Alexa Fluor633 (5.0 μg/ml: for membrane staining) for 30 min and 4',6-diamidino-2-phenylindole (for nuclear staining) for 10 min. The tissue explants were then washed once and mounted on slides to observe under Nikon A1R confocal microscope.

### HEK-293 cell culture and transfection

HEK-293 cells were cultured in complete DMEM media (with 10% fetal bovine serum, 100 units/ml penicillin, and 0.1 mg/ml streptomycin). About 2 × 10^5^ cells/well were plated in 6-well plates. After the cells reached 70% confluency, they were transfected with plasmids for pCMV3-cMyc-ATGL (Sino biological), p-mcherryC1-CGI-58-FLAG (cloned manually), or p-mcherryC1-CIDEC-HA (cloned manually) using lipofectamine 2000 (Invitrogen). Manufacturers’ protocol was exactly followed for transfection. Post 36 h, cell lysate was collected, freeze-thawed, and the whole cell lysate was used to perform an ATGL activity assay.

### Cloning mouse CGI-58

Mouse CGI-58 was PCR amplified using cDNA from 3T3-L1 adipocytes. High fidelity polymerase from Takara (catalog no.: #R045) was used at the following PCR conditions to amplify mouse CGI-58 with a 2×FLAG tag at the C terminus. PCR conditions are as follows: initial denaturation at 95 °C for 1 min, subsequent denaturation at 98 °C for 7 s, annealing at 58 °C for 15 s, and extension at 72 °C for 40 s for a total number of 35 cycles using primers, (forward CGAGCTCAAGCTTATGAAAGCGATGGCGGCGGAGGAGG and reverse ACGCGGATCCTCACTTGTCGTCATCGTCTTTGTAGTCCTTGTCGTCATCGTCTTTGTAGTC). PCR product was double digested with Hind-III and Bam-H1 and gel purified. The pEGFP-N1 plasmid obtained from Addgene (catalog no.: #54767) was also double digested with Hind-III and Bam-HI and gel purified. A purified PCR fragment was inserted into the HindIII–BamH1 digested plasmid and the positive clones were selected on kanamycin. Gene expression was confirmed by Western blotting using an anti-FLAG antibody as well as the anti-CGI-58 antibody from Santa Cruz Biotech. A single band at the expected size of ∼45 kDa was observed using both antibodies, confirming robust protein expression.

### ATGL activity assay

ATGL activity assay was performed as described earlier (9). Briefly, ATGL (Sino Biological, catalog no.: MG52782-CM), CGI-58-FLAG (cloning as described earlier), or CIDEC-FLAG (Sino Biological, catalog no.: HG11420-NF) were transfected into HEK-293 cells. The cell lysates were purified by centrifugation, quantified, and used in the ATGL activity assay. The assay was performed by addition of either alone or combination of 80 μg ATGL, 40 μg CGI-58, and/or 40 μg CIDEC. The final volume of ATGL activity buffer (250 mM sucrose, 1 mM EDTA, 1 mM DTT, protease inhibitor, a phosphatase inhibitor, 0.005 % BSA; pH 7.0) was 90 μl. Ten microliters of 10 mM glyceryl trilinoleate (final concentration 1 mM) was added to the aforementioned ATGL activity buffer. The complete reaction mixture (total 100 μl) volume was incubated for 30 min at 37 °C. For each measurement, 20 μl of the reaction mixture was used to assess glycerol using free glycerol reagent (Sigma).

### RNA-seq analysis

Visceral adipose tissue from six animals in each group were used, and the RNA samples were pooled in two sets of three animals each, making up to two sets of pooled samples in floxed and two sets of pooled samples in the Ad-hFSP27tg group. Total RNA was extracted from the tissues of biological replicates using Trizol reagent (Life Technologies Inc). Subsequently, the total RNA was treated with DNAseI (Qiagen) to remove coisolated genomic DNA and purified using RNeasy MinElute Cleanup Kit (Qiagen). The total RNA's quality and quantity were analyzed using Agilent 2100 Bioanalyzer and Qubit 4 Fluorometer (Invitrogen), respectively. The RNA-seq libraries were prepared using NEBNext Ultra II RNA Library Prep Kit according to the manufacturer's protocol (NEB). The mRNAs were enriched using Oligo (dT) beads, and subsequently, they were fragmented into shorter fragments using fragmentation buffer. The first-strand cDNA was synthesized from the fragmented mRNA using random hexamer primers and later converted into double-strand cDNA. The resulted double-strand cDNAs were end-repaired and added with Illumina sequencing adapters. The adapter-ligated libraries were amplified using sequencing primers for the enrichment. The library's quality and insert size were determined using a bioanalyzer (Invitrogen), and the library was quantified using a Qubit fluorometer (Invitrogen). The library was diluted to 4 nM concentration and sequenced using Illumina's NextSeq 500 platform with paired-end sequencing chemistry. The resulted image files in the BCL format were converted to FASTQ with 2 × 75 bp reads using the bcl2fastq tool (Illumina). The sequencing adapters and the low-quality reads (Phred score QV <30) were removed using a read trimming tool Trimmomatic (61). The quality-filtered reads were mapped to the mouse (strain C57BL/6J) reference genome (https://www.ncbi.nlm.nih.gov/assembly/GCF_000001635.20/) using STAR RNA-seq aligner (62) to generate BAM alignment. The read count table was generated from the BAM alignment file and general feature format of genome annotation using the HTSeq R package (63). The DEGs among different experimental pairwise combinations were identified using the edgeR package (64). The DEGs were filtered based on the minimum log2FoldChange of 1 and false discovery rate of 0.05. Gene Annotation, GO Enrichment analyses were performed using Omicsbox (https://www.biobam.com/omicsbox/) and the MGI database (http://www.informatics.jax.org/). Gene Network analysis was done using Cytoscape (https://cytoscape.org/) and STRING database (https://string-db.org/). Pathway mapping was performed using KOBAS (65) and the KEGG database.

### Confocal microscopy

Confocal microscopy was performed on Nikon A1R (Nikon) confocal microscope using 60 × oil immersion objective. Images were processed using Nikon software.

### Statistical analysis

Data were expressed in mean ± SEM, unless otherwise mentioned. For statistical significance, student’s *t* test was used to assess the significance between the two groups. One-way or two-way ANOVA was used to analyze significance between multiple groups followed by specific post hoc analysis as depicted in figure legends. *p* values of ∗*p* < 0.05, ∗∗*p* < 0.01, ∗∗∗*p* < 0.001 were used to determine statistical significance. Data were analyzed by GraphPad Prism (version 5.01, GraphPad Software Inc).

### Antibodies list

#### For human studies

CIDEC: Rabbit (1:1000), Thermofisher (PA5-30793), (species: Human)

ATGL: Rabbit (1:1000), Thermofisher (PA1-16974), (species: Human, Mouse)

HSL: Rabbit (1:1000) Abcam (ab45422), (species: Human)

#### For mouse studies

FSP27: Rabbit (1:1000) Abcam (ab198204), (species: Human, Mouse)

β-Tubulin: Rabbit (1:1000) Cell Signaling Technology (CST-2146), (species: Human, Mouse)

phospho-AKT (Ser473): Rabbit (1:1000) Cell Signaling Technology (CST-9271), (species: Human, Mouse)

AKT: Rabbit (1:1000) Cell Signaling Technology (CST-9272), (species: Human, Mouse)

ATGL: Rabbit (1:1000) Cell Signaling Technology (CST-2138), (species: Human, Mouse)

CGI-58: Rabbit (1:1000) Biovision (3901-30T), (species: Human, Mouse)

Rabbit (DA1E) mAb IgG XP Isotype Control: Rabbit (1:500) Cell Signaling Technology (CST-3900), (species: All)

Anti-rabbit IgG, HRP-linked antibody: HRP-linked secondary antibody Goat (1:5000)

Cell Signaling Technology (CST-7074), (species: All)

## Primers

Human CIDEC (for genotyping): TCAGCTTGTACAGATCAAACGTAAC (forward); GGATGTGCCATGTGAGTCTG (reverse)

Mouse Fsp27 (for genotyping): AGCTTGGGTCGGAGAAACAATG (forward); GTCCATCCTTGTCAGTTGGA (reverse)

Adipo Cre (for genotyping): GGATGTGCCATGTGAGTCTG (forward); CGGACAGAAGCATTTTCCA (reverse)

Human CIDEC (for qRT-PCR): TCCAGCTGACAAGGATGGAATACG (forward); CCTCAAGACTGTAAGCCATGATGC (reverse)

## Data availablity

All data are included in the article.

## Supporting information

The article contains supporting information.

## Conflict of interest

The authors declare that they have no conflicts of interest with the contents of this article.
