# Supplementary material for: Human CIDEC transgene improves lipid metabolism and protects against high-fat diet–induced glucose intolerance in mice
Source: J Biol Chem. 2022 Aug 11;298(9):102347. doi: 10.1016/j.jbc.2022.102347 (PMC9472082; doi:10.1016/j.jbc.2022.102347)
Supplement: Supplemental Table S1 [file mmc1.docx]

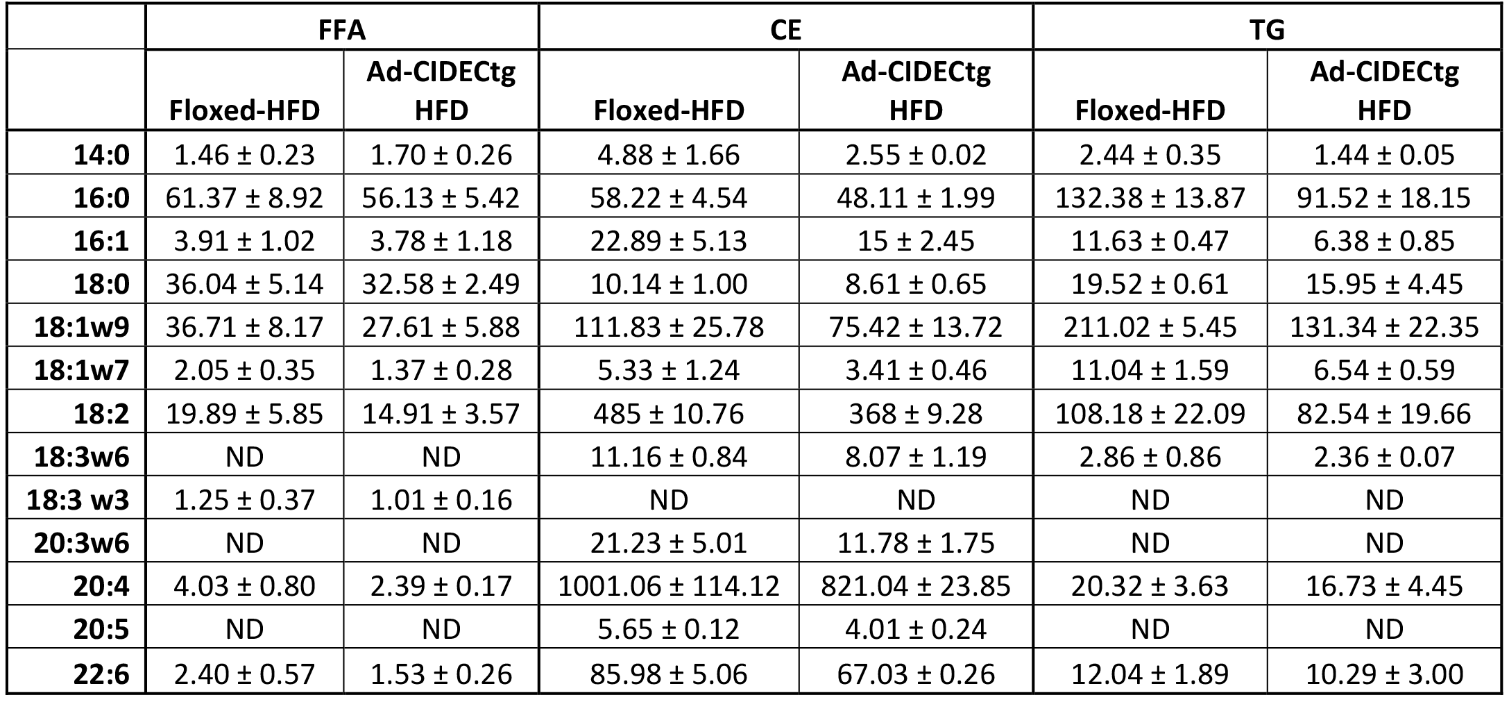


**Supplementary Table 1:** Detailed fatty acid distribution within free fatty acids, cholesteryl esters, and triglyceride fractions (µg/ml) in HFD-fed floxed-controls and Ad-CIDECtg mice.
