# Supplementary material for: Human CIDEC transgene improves lipid metabolism and protects against high-fat diet–induced glucose intolerance in mice
Source: J Biol Chem. 2022 Aug 11;298(9):102347. doi: 10.1016/j.jbc.2022.102347 (PMC9472082; doi:10.1016/j.jbc.2022.102347)
Supplement: Supplemental Figures S1–S6 [file mmc2.docx]

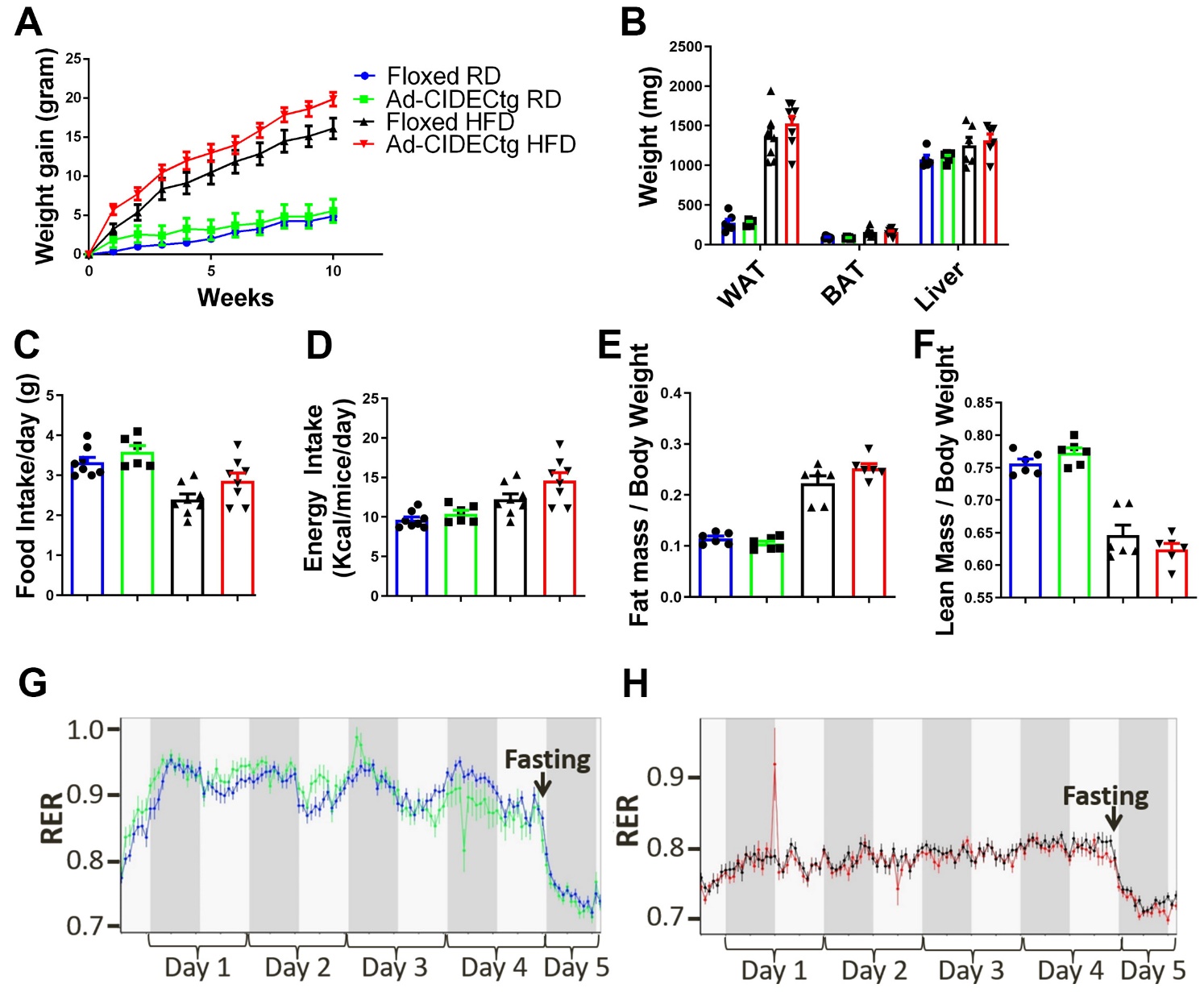


**Supplemental Figure SF1, Gupta et al.**


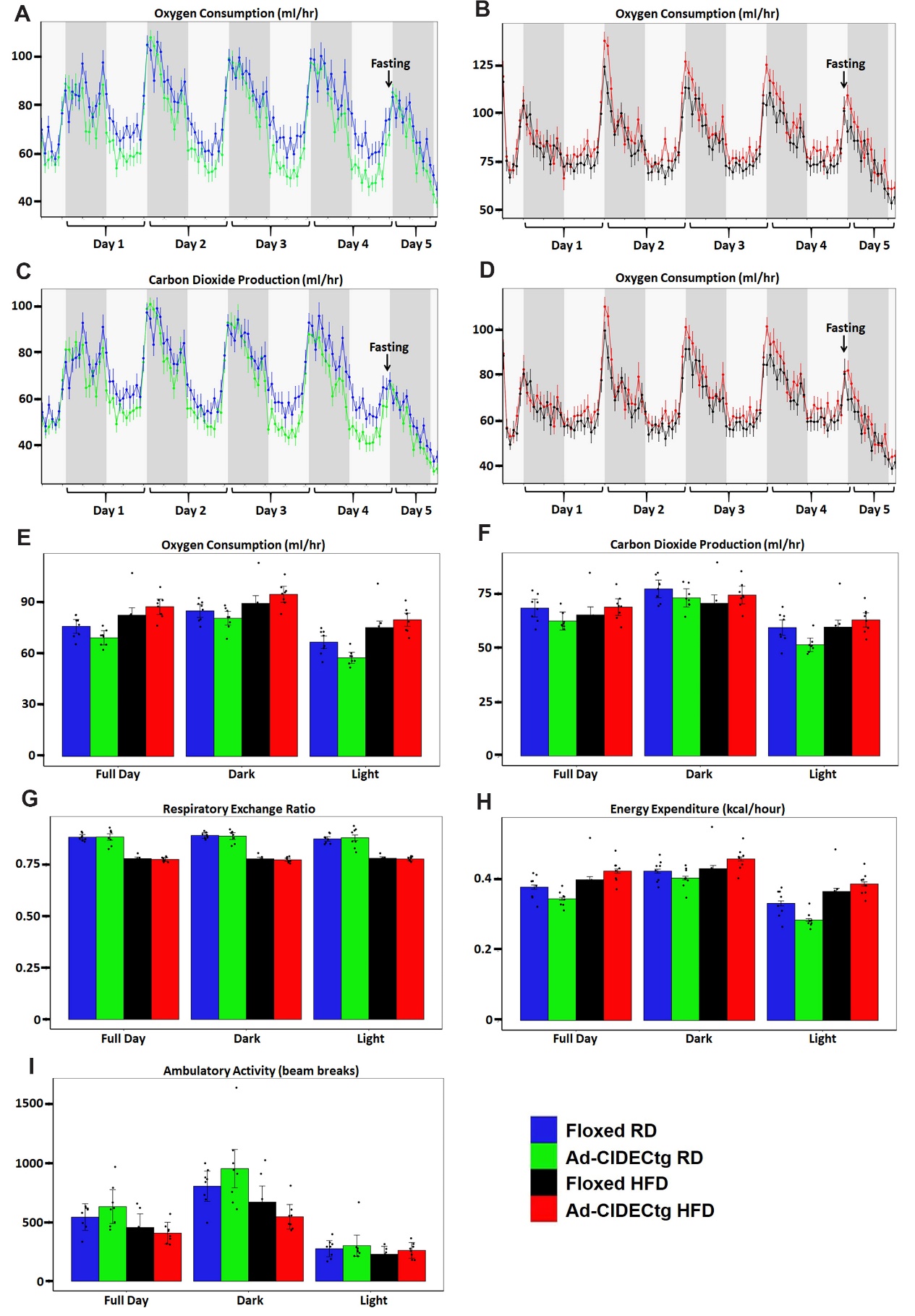


**Supplemental Figure SF2, Gupta et al.**


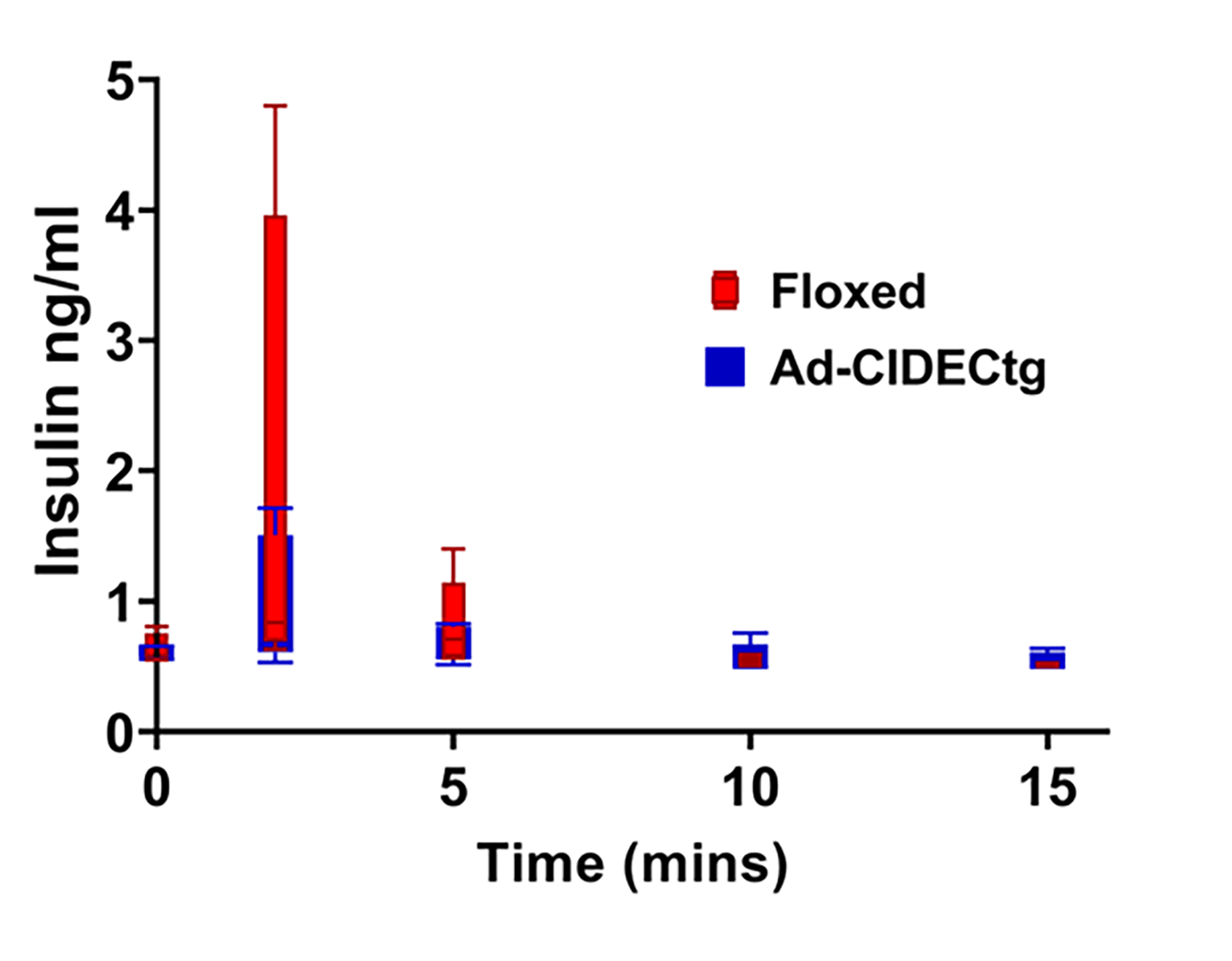


**Supplemental Figure SF3, Gupta et al.**


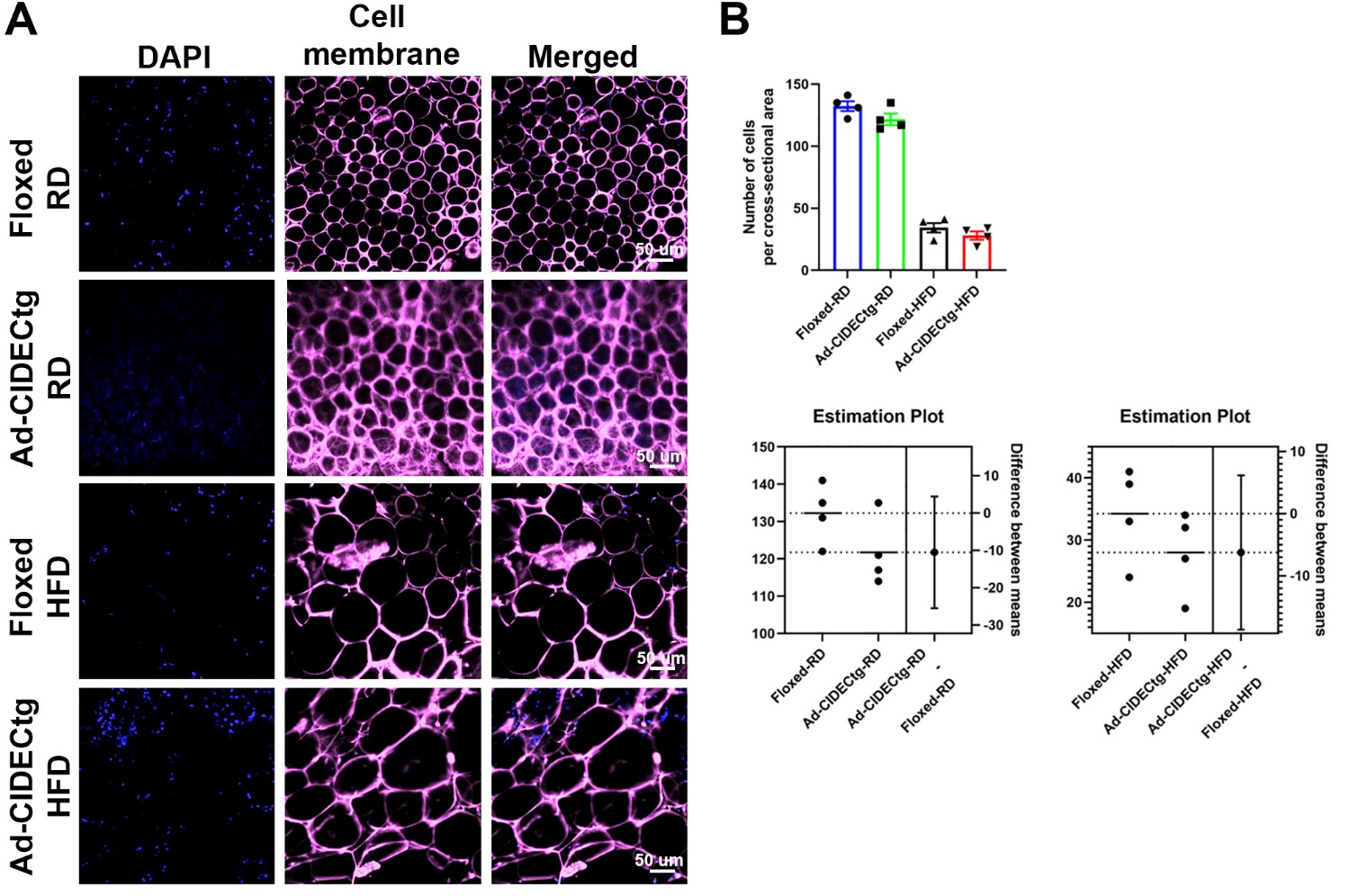


**Supplemental Figure SF4, Gupta et al.**


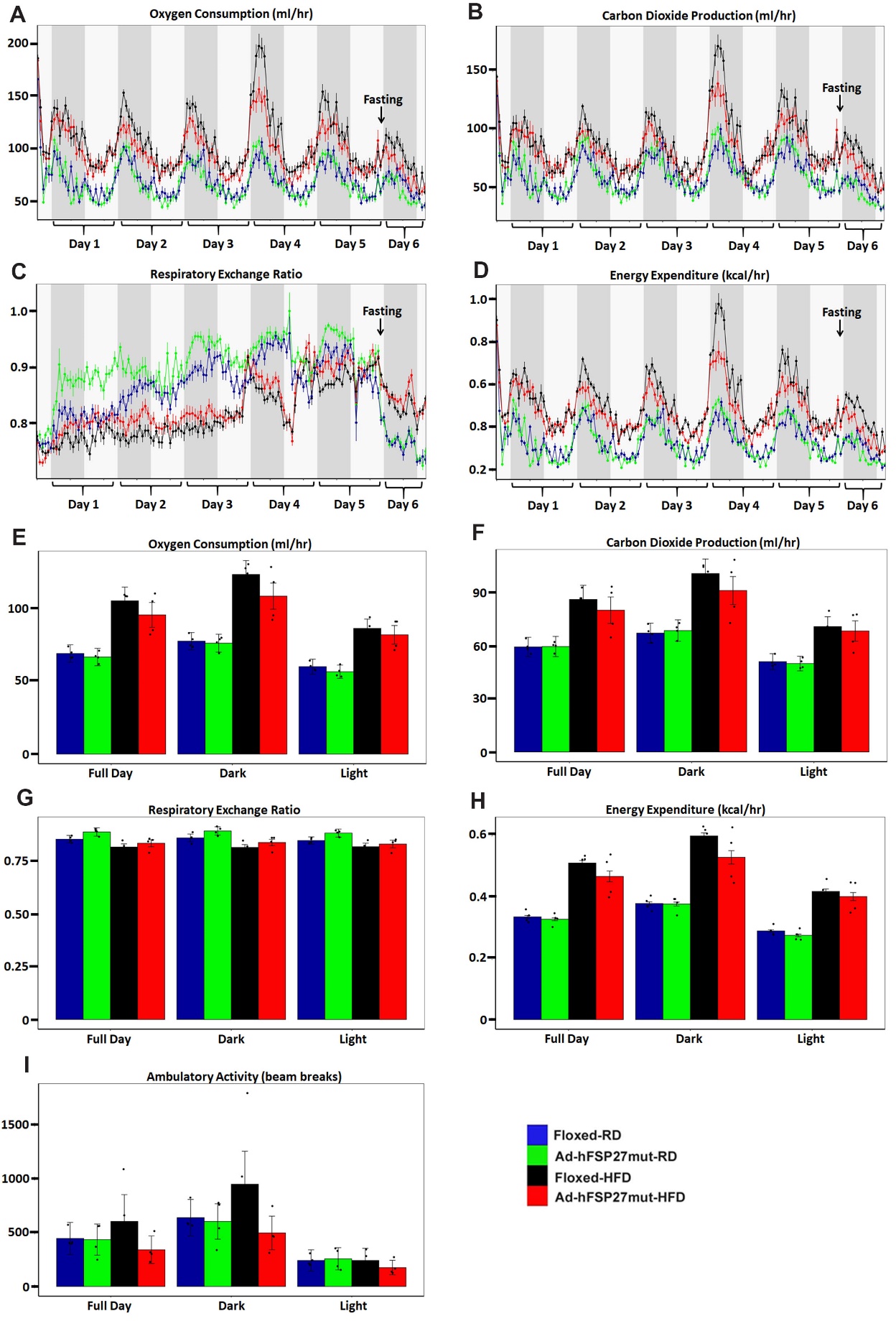


**Supplemental Figure SF5, Gupta et al.**


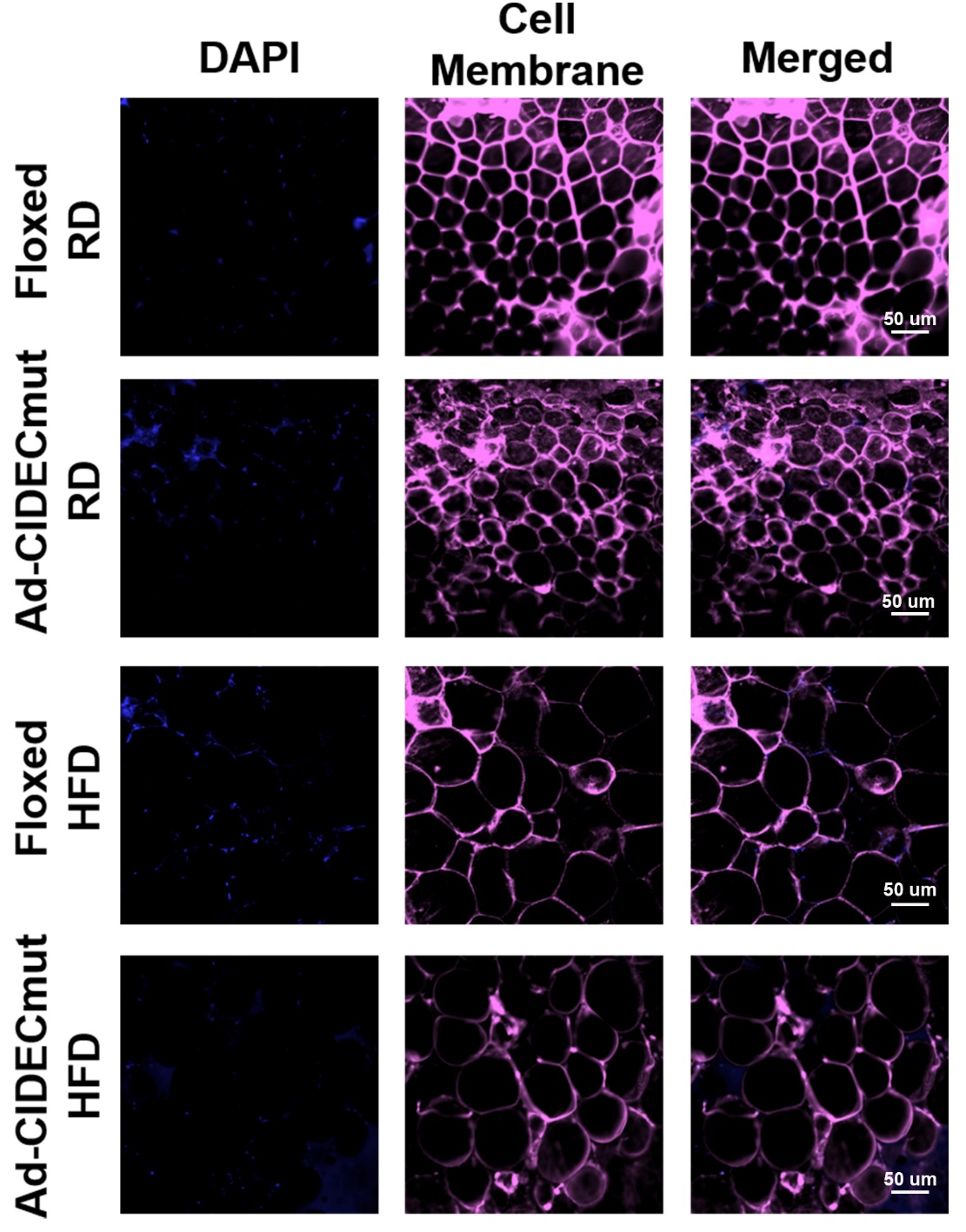


**Supplemental Figure SF6, Gupta et al.**

**Supplementary Figure SF1:** **Metabolic phenotyping of Ad-CIDECtg. (A)** Weight gain of floxed and Ad-CIDECtg mice fed either a regular or high-fat diet. Two-way ANOVA followed by Bonferroni post hoc analysis was performed for statistical analysis. **(B)** Organ weight of floxed and Ad-CIDECtg mice. **(C)** Food intake and **(D)** Energy intake of floxed and Ad-CIDECtg mice. Body composition measurement of mice measuring **(E)** Fat mass or **(F)** Lean mass normalized to body weight. In B-F, blue, green, black, and red bars represent Floxed RD, Ad-CIDECtg RD, Floxed HFD, and Ad-CIDECtg HFD, respectively. The respiratory exchange ratio was analyzed in floxed and Ad-CIDECtg mice upon both regular chow **(G)** and HFD feeding **(H)**. Color codes for SF1 (C-F) are like SF1B. Data are represented as mean ± SEM. One-way ANOVA was performed to analyze the significance between multiple groups.

**Supplementary Figure SF2:** **Indirect calorimetry analysis of floxed and Ad-CIDECtg mice fed regular chow or high-fat diet.** **(A)** Oxygen consumption rate in floxed and h-CIDECtg mice fed a regular chow diet. **(B)** Oxygen consumption rate in floxed and h-CIDECtg mice fed a HFD. **(C)** Carbon dioxide production rate in floxed and h-CIDECtg mice fed a regular diet. **(D)** Carbon dioxide production rate in floxed and h-CIDECtg mice fed a HFD. **(E)** Absolute oxygen consumption rate of floxed and Ad-CIDECtg mice fed regular chow and HFD over the full day and light and dark cycles. **(F)** Absolute carbon dioxide production rate of floxed and Ad-CIDECtg mice fed regular chow and HFD over the full day and light and dark cycles. **(G)** The respiratory exchange ratio of floxed and Ad-CIDECtg mice on regular chow and high-fat diet over the full day and light and dark cycles. **(H)** Energy expenditure of floxed and Ad-CIDECtg mice on regular chow and HFD over the full day and light and dark cycles. **(I)** The ambulatory activity of floxed and Ad-CIDECtg mice on regular chow and HFD over the full day and light and dark cycles.

**Supplementary Figure SF3: Basal and glucose-stimulated Insulin levels in HFD-fed floxed-controls and Ad-CIDECtg mice.** Mice were fasted overnight and blood was withdrawn to ­­­measure basal levels of insulin. IP injections of glucose were given as described in GTT assay and blood was withdrawn at various time points as indicated and insulin levels were measured using mouse ultrasensitive insulin ELISA kit from Alpco (Cat no 80-INSMSU-E01; n=6 per group). Data are represented as mean ± SEM. For statistical analysis, a student’s t-test was applied to compare the significance between the two groups.

**Supplementary Figure SF4: Adipocyte morphology in adipose tissue of floxed and Ad-CIDECtg mice. (A)** Adipocyte morphology in the perigonadal white adipose tissue of floxed and Ad-CIDECtg mice fed regular chow or HFD. **(B)** Cell number per cross-sectional area was estimated in perigonadal adipose tissue from floxed or Ad-CIDECtg mice fed either regular-chow or high-fat diet. No statistical significance was observed, with p-value of 0.1371 between regular-chow diet groups, and 0.2640 between HFD-fed groups. Student’s t-test was performed to assess the significance between the two groups. At the bottom of panel B, the left box shows an estimation plot depicting the distribution of data along mean values, where the dotted line is the mean of the group, and the dots show the distribution of data along mean values. The right-side smaller box shows a difference between the mean values of the groups at 95% confidence interval.

**Supplementary Figure SF5:** **Indirect calorimetry analysis of floxed and Ad-CIDECmut mice fed regular chow and HFD.** **(A)** Oxygen consumption rate in floxed and Ad-CIDECmut mice fed regular chow and HFD. **(B)** Carbon dioxide production rate in floxed and Ad-CIDECmut mice fed regular chow and high-fat diet. **(C)** Respiratory exchange ratio in floxed and Ad-CIDECmut mice fed regular chow and high-fat diet. **(D)** Energy expenditure in floxed and Ad-CIDECmut mice fed regular chow and high-fat diet. **(E)** Absolute oxygen consumption rate in floxed and Ad-CIDECmut mice fed regular chow and high-fat diet during the full day and light and dark cycles. **(F)** Absolute carbon dioxide production rate in floxed and Ad-CIDECmut mice fed regular chow and HFD during the full day and light and dark cycles. **(G)** Absolute respiratory exchange ratio in floxed and Ad-CIDECmut mice fed regular chow and HFD during the full day and light and dark cycles. **(H)** Absolute energy expenditure in floxed and Ad-CIDECmut mice fed regular chow and HFD during the full day and light and dark cycles. **(I)** Ambulatory activity in floxed and Ad-CIDECmut mice fed regular chow and HFD during the full day and light and dark cycles.

**Supplementary Figure SF6: Adipose tissue images from floxed and Ad-CIDECmut mice.** Adipocyte morphology in perigonadal white adipose tissue of floxed and Ad-CIDECmut mice either fed regular chow or HFD.
